# Representation of women in scientific subjects: overview of systematic reviews investigating career progress in academic publishing with a focus on mental health

**DOI:** 10.1192/bjo.2024.820

**Published:** 2025-03-12

**Authors:** Til Wykes, Sanchita Garg, Daniel Stahl, Ayse Kostem, Emma Wilson-Lemoine

**Affiliations:** Institute of Psychiatry, Psychology and Neuroscience, King’s College London, UK; South London and Maudsley NHS Foundation Trust, London, UK

**Keywords:** Career, gender, glass ceiling, sex, Science, Technology and Engineering

## Abstract

**Background:**

Women’s authorship position in science, technology, engineering, mathematics and medicine research reflects career progression, especially the transition from first to last (usually senior) author. Employment of women in mental health sciences has increased, and so should have had an impact on the change to senior author position.

**Aims:**

To identify if first or last women’s authorship has changed, and mental health has better representation.

**Method:**

We investigated women’s authorship position in a systematic review and meta-analyses, following PRISMA guidelines and using random-effects regression analyses.

**Results:**

We identified 149 studies with sampling periods from 1975 to 2020 (excluding potential COVID-19 pandemic effects) that showed a large variation of women authors, and found an average proportion for first (26.2%) and last (16.1%) author position. In mental health (psychology and psychiatry), there was a higher representation, with 40% first author and 36.7% last author position, whereas medicine was 25.9% and 19.5%, respectively. The rate of change for psychology and psychiatry women authors was also higher every 10 years: 8.56% (95% CI 6.44–10.69%) for first and 6.86% (95% CI 4.57–9.15%) for last author, and rate was 2.35% higher for first author and 2.65% higher for last author than in medicine. Different methods of classifying gender and identification method did not affect our results.

**Conclusions:**

Although mental health topics seem to fare better, our comprehensive review highlighted that the proportions of women first compared with last authors shows the same leaky pipeline as in other analyses, so we cannot be complacent about gender equality and career progression.

Since the start of mandatory gender pay gap reporting in 2017, we know that around 80% of employers in the UK pay men more than women.^1^ In some occupations, the gender pay gap has also worsened since mandatory reporting, and one of the worst sectors is higher education, where it has increased to 22%.^2^ Transparency, at least for some occupations, has not had the desired effect, and promotion prospects are likely to feature as a reason for this gap. UK universities have been encouraged to apply for Athena Swan awards, which emphasise how to support and transform gender equality within higher education and university research;^2^ however, this does not seem to have moved the dial in terms of pay gaps.

A barrier to promotion, and subsequently higher pay, is the ‘publish or perish’ principle adopted across the world.^3–6^ Women therefore need to be visible in the research arena as authors of peer-reviewed academic publications. Although the number of women authors in science, technology, engineering, mathematics and medicine (STEMM) is generally low, there is a consensus that the proportion has steadily increased over the past few decades.^7–9^ In mental health science, the proportion of women employed is generally higher than in other STEMM fields. In UK psychiatry, the number has doubled over 10 years (up to 2019), to 40%, and in the USA, it is 42%.^10,11^ For psychology, in the USA, the employment rate is 53%, and in the UK, 63% of lecturers are women, although senior university positions are held by only 31%.^12,13^

Author position in empirical topics is often an indicator of the prominence of women authors, and an indicator of potential promotion. By convention, first authors tend to be more junior and last authors are mostly (but not universally) considered as more senior.^14,15^ Although this may vary between fields because of cultural practices, analysing author positions can reveal how quickly women are advancing in their careers. The increased employment in mental health fields may have influenced the role of women authors in publications, and therefore herald a turning point. If not, then this needs immediate attention, and should be a focus for universities in their development of promotion strategies to support women’s career progression.

There are some subtleties to understanding the numbers produced in existing reviews. Methodological differences exist, such as amalgamating studies using different approaches to determine the author gender binary of men and women.^16^ Manual approaches involve researchers assigning gender based on individual judgements, and computational approaches assign gender by referring to online databases containing names. These approaches bring their own unique limitations. For example, algorithm-based approaches often disproportionally misclassify stereotypically non-Western names because of an overreliance on exclusively Western databases, as well as the misclassification of androgynous names.^17^ For instance, one author of this review would not be classified as a woman based on her given name, with an algorithmic approach.^18^

By analysing published systematic reviews, we can examine the effect of scientific topic with controls for methodological differences in existing studies, and so, provide an unconfounded baseline against which the success of interventions to improve women’s career trajectories can be measured. We have specifically chosen to investigate and compare the effects in psychology and psychiatry with other STEMM disciplines, to understand whether increased employment has had any effect. Our objectives are to (a) assess the gender distribution among first and last authorship positions over time; (b) investigate the gender representation across different scientific disciplines to identify discipline specific challenges for female career advancement; (c) evaluate whether author classification methods affect the results; (d) explore the relationship with career progression over time, focusing on the transition from junior to senior academic positions; and (e) establish a methodologically sound baseline of women’s representation in academia, which allows assessment of future interventions to support women’s career progression, especially in psychology and psychiatry.

## Method

### Literature search

We followed Preferred Reporting Items for Systematic Reviews and Meta-Analyses (PRISMA) guidelines.^19^ The protocol was registered in OSF Registries on 15 January 2020 (registration DOI: 10.17605/OSF.IO/BYJPD). Ethical approval was not needed as we used publicly available data.

All articles and reviews were retrieved from Ovid Medline(R), EMBASE, PsycINFO, BASE, Web of Science and CINAHL from inception to 29 January 2020 when the data were first extracted, and then updated on 25 October 2021. To ensure consistency and comparability, our analysis focused on studies published before the onset of the COVID-19 pandemic. The impact of COVID-19 is widely acknowledged as a disruptive event that can affect temporal trends, and our approach guarantees a more coherent and uninterrupted examination of trends and outcomes over time. The search strategy was: (‘representation’ OR ‘proportion’) AND (‘women’ OR ‘female’) AND (‘authorship’ OR ‘authors’) AND ‘publications.’ Additional papers were identified via reference lists of relevant articles and reviews. Studies were eligible if they were peer-reviewed original articles or reviews; had a retrospective, observational or analytical study design; reported the proportion of women authors in science publications and were in English.

Titles and abstracts were independently screened, and full texts were retrieved by three researchers (E.W.-L., S.G. and A.K.). Any screening differences were resolved via consultation. Data were extracted from the included studies and again agreed or validated by S.G., A.K. or E.W.-L. The items extracted were as follows:

- study characteristics: authorship, year;
- study focus: the discipline of publications based on categories provided by the studies, scope (i.e. number of journals included in the analysis), years sampled;
- methodology: method of identifying women (i.e. manual or computational), classification of gender or sex: whether gender, sex or both were the focus, actions taken if unable to determine author gender or sex;
- proportion of women in publications: percentages of women first authors, percentages of women last authors.

### Data analyses

We conducted an analysis of gender distribution among authors in identified systematic review publications, which is called an ‘overview of reviews’ analysis according to Cochrane.^20^ The primary outcome was the proportion of women as first or last author, defined as the number of women as the first or last author divided by the total number of publications assessed within each published survey. Women as first and last authors were analysed separately.

For articles that only provided data about the proportion of women as first or last author and in figures and not the tables or text (*n* = 39, 28.5%), the percentage was estimated from the figures. As studies often provided only total sample sizes, not sample sizes within each sampling time frame, we were not able to perform a standard meta-analysis, which requires sample sizes to calculate s.e. and weights. Instead, we employed the more appropriate random-effects meta-regression analysis, using the proportion of women authors as the outcome variable and considering the sampling period and potential confounders as independent variables. To account for repeated observations over time within a study, we included ‘study’ as a random effect in the model. Some publications presented data from different disciplines or journals separately. We modelled these data points as separate ‘studies’, and controlled for the dependency of studies within the same paper by including ‘publication’ as an additional random effect. A preliminary analysis showed that the ‘sampling period’ could be modelled as a linear term and needed to be included as a random slope. A random slope allows us to account for differences in how trends varied between studies. We used an unstructured covariance matrix, which means we allowed both the starting point (random intercept) and the trend (slope) to vary independently from one another. This gives the model more flexibility to fit the data from different studies without assuming a strict relationship between them, making the analyses more robust.

We assessed interactions with sampling year, but if these were not statistically significant, they were not included in subsequent analyses.

To investigate potential variations in the trajectory of seniority across scientific disciplines, we needed to merge meaningful disciplines into larger ones to ensure sufficient sample sizes for statistical analyses. For this merging, we took note of amalgamations made previously in the literature, and throughout we refer to journals, not the topic of the paper.

#### Analyses steps

In the first step, we looked at the changes in the proportion of women authors over the sample period. In the second step, we added the discipline of the study and assessed the interaction between discipline and sample period. In the last step, we also included how women authors were recorded (sex/gender/both) and the classification method (algorithm/manual/both) to investigate if this influences the trajectory. Because missing data are assumed to be missing complete at random, a complete-case analyses at each step was performed. Analysis estimates are presented with 95% confidence intervals.

#### Sensitivity analyses

If the sampling period was presented for more than a year, we included the mean of the sampling period. We performed a sensitivity analysis by rerunning the analyses with interval sampling length (≤5 years *v*. >5 years) as a covariate to assess if longer sampling periods influence the parameter estimates. A second sensitivity analysis included type of data extraction (tables/text versus derived from figures) as a covariate.

## Results

### Scope of review

**Fig. 1:**
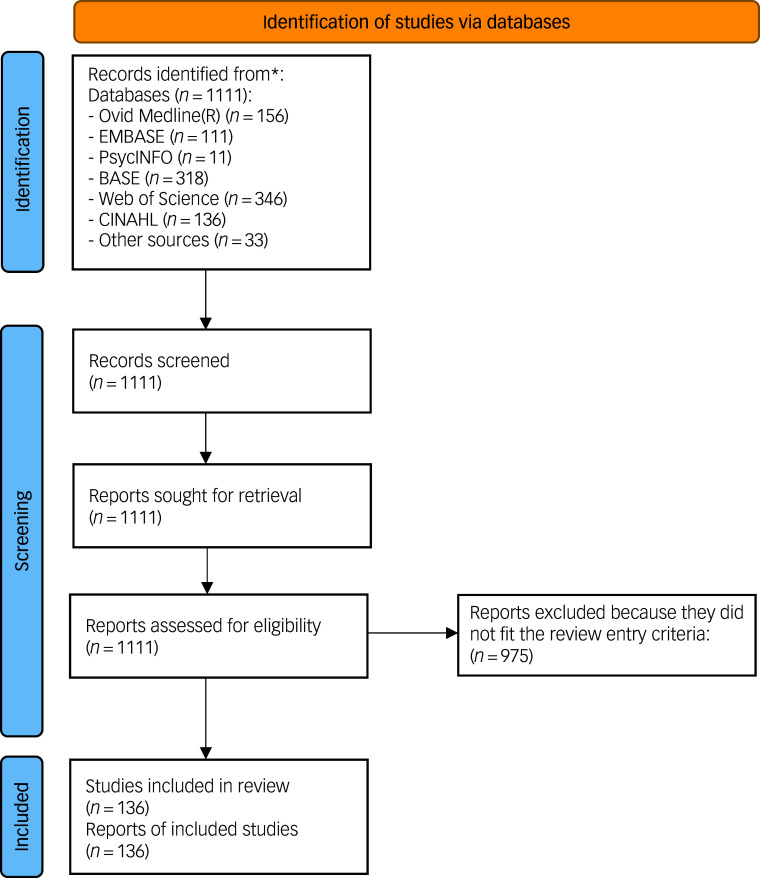
PRISMA flowchart.^21^ *Consider, if feasible to do so, reporting the number of records identified from each database or register searched (rather than the total number across all databases/registers). If automation tools were used, indicate how many records were excluded by a human and how many were excluded by automation tools. This work is licensed under CC BY 4.0 (https://creativecommons.org/licenses/by/4.0/).

Figure 1 shows the PRISMA flowchart for this review. A total of 136 survey studies met the inclusion criteria and were included in the systematic overview review, with a total of 885 sampling periods ranging from 1 to 21 years (mean 6.5, s.d. 11.0). Although the sampling periods ranged from 1910 to 2020, numbers in earlier years were small, so we removed any samples before 1975 (*n* = 34). Six papers presented more than one independent study, resulting in a total of 149 studies. Supplementary Table 1 provides all of the data for the studies included in this review.

Studies were across seven disciplines (engineering *n* = 1, medicine *n* = 119, psychology and psychiatry *n* = 16, natural science *n* = 8, social science *n* = 2, information science *n* = 1 and multidisciplinary, defined as more than one discipline studied *n* = 2), and these papers were published between 1996 and 2021. The disciplines represented were grouped into three types: medicine (*n* = 119); psychology and psychiatry and social sciences (*n* = 18); and engineering, natural science, information science and multidisciplinary (*n* = 12). The small number of published surveys for the latter two limits the interpretation of non-significant results.

### Potential confounders

Of the 136 articles studies, 77 (56.6%) used manual methods, 25% (*n* = 34) used computational methods and 15.4% (*n* = 21) used both; four (2.9%) did not provide enough information to determine their approach. Papers that incorporated algorithms were published, on average, 4.1 years later than those solely relying on manual methods (*z* = 2.78, *P* = 0.005), as might be expected with the growth of publications and technology allowing for short cuts. ‘Gender’ was referred to by 92 (67.6%) studies, seven (5.1%) used ‘sex’, and 37 (27.2%) used both ‘gender’ and ‘sex’. Publications that exclusively used the term ‘gender’ tended to be published later compared with those that used ‘sex’ (5.2 years, *P* = 0.095) and those using both ‘gender’ and ‘sex’ (4.0 years, *P* = 0.013).

### Sensitivity analyses

We used the mean year for the data analyses when assessing the relationship between the year of data collection and the proportion of women first or last authors. Sufficient data for regression analyses were provided for 136 (100%) women as first author and 115 (86.1%) for last author. This number did not change after dropping sampling periods before 1975.

**Fig. 2:**
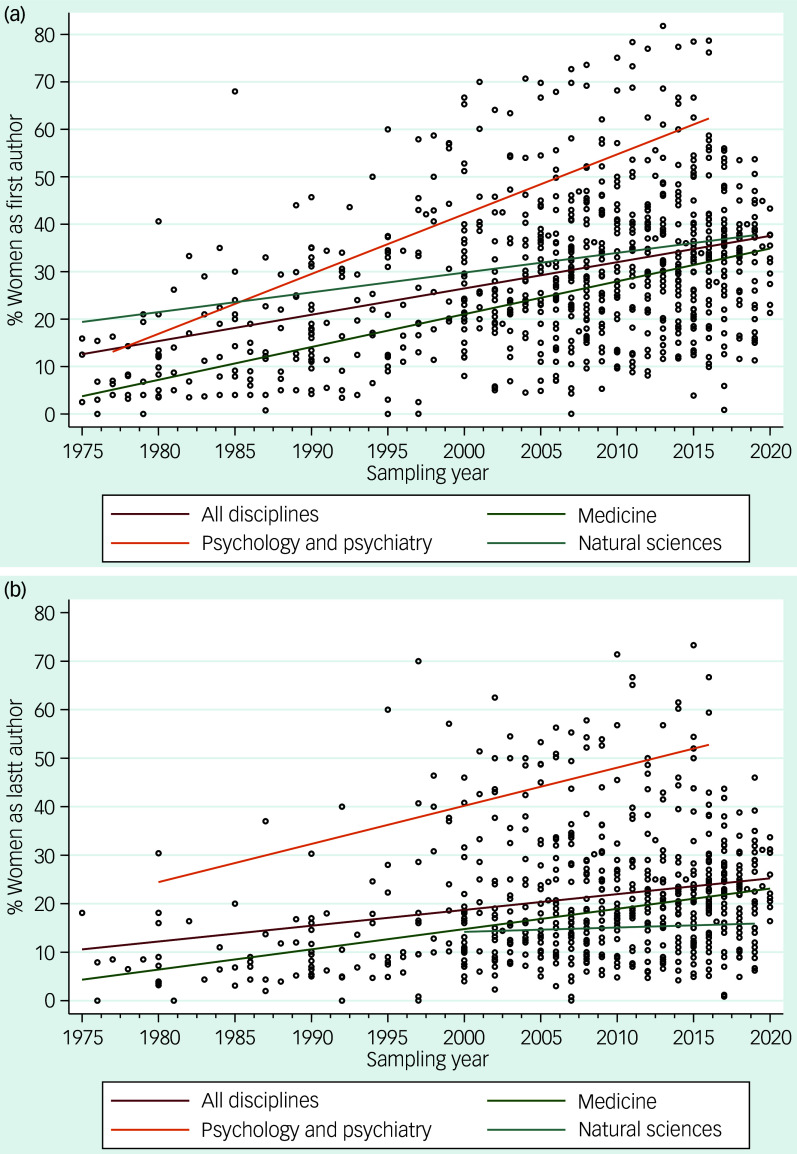
Scatter plots of the relationship between sampling time on the *x*-axis and percentage of women as (a) first and (b) last authors in publications. A linear trendline is shown in both figures for all disciplines together, and for medicine, psychology and psychiatry, and social sciences separately. Sample size was too small for other disciplines to plot trend lines.

Figure 2 shows the scatter plots describing the relationship between sampling time and the percentage of women as first and last authors. For the analysis involving all data published before 1975, please refer to Supplementary Fig. 1.

### First authors

A random-effects regression analysis reveals that the proportion of women as first authors increases by 6.4% for every 10 years (*b* = 0.641 (95% CI 0.556–0.725), *z* = 14.92, *P* < 0.0001, *n* = 136, with 846 observational time periods). When discipline was added to the model, the rate of change over time appeared similar across disciplines, as the interaction between time and discipline was not significant (*P* = 0.19). For easier interpretation, we removed the non-significant interaction between time and discipline, revealing significant differences between disciplines. The proportion of women as first authors in psychology and psychiatry was 15.0% higher (95% CI 9.49–20.46%, *z* = 5.35, *P* < 0.001) compared with medicine, and 8.80% higher (95% CI 0.76–16.83%, *z* = 2.15, *P* = 0.032) compared with engineering, natural science and information science. The proportion of women as first authors in engineering, natural science and information science was 6.17% higher (95% CI −0.35 to 12.7%, *z* = 1.86, *P* = 0.063) compared with medicine. In 2020, the estimated proportions of women as first authors were as follows: psychiatry and psychology 40.8% (95% CI 35.7–46.0%); medicine 25.9% (95% CI 23.9–27.9%); and engineering, natural science and information science 32.0% (95% CI 25.8–38.3%).

An exploratory analysis also revealed that psychiatry and psychology experienced a steeper increase in women first authors compared with medicine (see Fig. 2). Specifically, the rate of change for women first authors in psychology and psychiatry increased by 8.56% every 10 years (95% CI 6.44–10.69%), and the rate was 2.35% (95% CI 0.03–4.67%), *z* = 1.99, *P* = 0.047) higher than that for medicine, which was 6.21% (95% CI 5.28–7.14%). This interaction was significant (slope difference: 0.235, 95% CI 0.003–0.467, *z* = 1.99, *P* = 0.047). Comparisons with the natural sciences were not carried out because of the small sample size.

There were no significant differences in women first author contribution when the paper classified authors by gender, sex or sex and gender (Wald *χ* ^2^(2) = 0.43, *P* = 0.80); no effects of the classification method (algorithm or manual or both: Wald *χ* ^2^(2) = 1.15, *P* = 0.56) and no interactions with sampling time with either sex and gender classification (*P* = 0.95) or classification method (*P* = 0.48).

### Last authors

The analysis of women as last authors reveals that the proportion increases by 4.22% for every 10 years (95% CI 3.61–4.91%, *z* = 12.36, *P* < 0.0001, *n* = 115, with 675 observational time periods). When discipline was added to the model, the rate of change over time appeared similar across disciplines, as the interaction between time and discipline was not significant (*P* = 0.11). After the removing the non-significant interaction, again significant differences were observed between disciplines. The proportion of women last authors in psychology and psychiatry was 17.3% higher (95% CI 11.4–23.14%, *z* = 5.75, *P* < 0.001) compared with medicine, and 13.0% higher (95% CI 4.0–22.0%, *z* = 2.84, *P* = 0.004) compared with engineering, natural science and information science. There was no significant difference between engineering, natural science and information science and medicine (mean difference: 4.2% (95% CI −2.9 to 11.4%, *z* = 1.16, *P* = 0.25). In 2020, the estimated proportions of women as last authors were as follows: psychiatry and psychology 36.7% (95% CI 31.0–42.5%); medicine 19.5% (95% CI 17.7–21.2%); and engineering, natural science and information science 23.7 (95% CI 16.7–30.7%).

An exploratory analysis also revealed that psychiatry and psychology experienced a steeper increase in women’s representation as last authors compared with medicine. Specifically, the rate of change for women last authors in psychology and psychiatry increased by 6.86% every 10 years (95% CI 4.57–9.15%), and the rate was 2.65% (95% CI 0.26–5.03%, *z* = 2.18, *P* = 0.029) higher than that for medicine, which was 4.21% (95% CI 3.56–4.87%). Comparisons with the natural sciences or the combined engineering, natural science and information science group were not conducted because of its small sample size.

There were no significant differences in women last author contribution when the paper classified authors by gender, sex or sex and gender (*P* = 0.90), or by classification method (algorithm or manual), and did not influence the proportions of women as last authors (*P* = 0.63). Interactions with sampling time were also not significant (sex and gender classification: *P* = 0.52; classification method: *P* = 0.52).

### Model assumptions and sensitivity analyses

Visual inspection of the residuals did not reveal major violations of assumptions, except for a small number of outliers. Rerunning the analyses without the outliers did not result in any major changes to the conclusions. We also re-ran the analyses including type of data extraction (either tables/text or derived from figures) and interval length of sampling period (≤5 years *v*. >5 years) as covariates. Neither covariate altered the results and were not significant (type of data extraction: first author *P* = 0.20, last author *P* = 0.36; interval length: first author *P* = 0.13, last author *P* = 0.51).

## Discussion

### Does mental health science perform better than other STEMM topics?

The results from our regression analysis revealed that 29.7% of women are first authors of scientific publications between 1975 and 2020, with an increase of 6.4% in proportions every 10 years. The results for last authors were disappointing, but perhaps not a surprise: only 21.3% of last authors are women, and this increased by only 4.2% every 10 years. These levels are unaffected by the potential confounding effects of methods of classification and use of gender or sex. The results of slower growth in last author publications likely reflect poorer career progression, even in countries with higher rates of gender equality.^22,23^ So, although there has been some progress, increased representation as first author does not inevitably lead to more women as senior authors.

For journals in mental health, the figures improved, but not considerably, and the proportion of women first authors in 2020 was similar to their rate of employment (40.8%).^10^ This first author rate was consistently higher than medicine throughout the observation period, and the change in rate of women first authors was 8.56% every 10 years, which was also 2.35% larger than for medicine, perhaps because of the increase in employment of women academics in psychiatry. Similarly, the proportion for last authors in psychology and psychiatry was 36.7% in 2020, which was higher than medicine, and the rate of change was also higher at 6.86% (2.65% higher than medicine). Hart et al.^24^ found that women first authors in academic psychiatry approached 50% in 2018 but, like our study, found slower rates for the transition to senior authorship. For comparison, the 2024 issues of the *British Journal of Psychiatry* show that women first authors are at 47% and women last authors are at 32%, similar to the figures in our review. The increased representation in the academic community is still not reflected in similar improved rates of last author research output, suggesting a continuing ‘leaky pipeline’ of women not reaching senior levels.^25^ As a result, choosing any scientific field does not appear to boost your career chances of getting to the top, even when women are well represented at the earlier stages.

### Does the classification method affect the results?

Despite the potential limitations of classification methods, neither classification using gender and/or sex, nor type of classification method (i.e. assigning gender/sex by algorithm or manually), influenced the proportions of first or last authors, in this meta-analysis. Although the methods did not affect the actual proportions for past papers, it is still important to consider standardisation for future work, to avoid classification inconsistencies. This is especially important given that there is an increase in non-Western authors of science publications, and it may be difficult to capture sex or gender given the current algorithmic methods.

### What barriers remain that affect women’s representation in STEMM publications?

The barriers are general and affect all women irrespective of choice of scientific topic, although there may be some nuances that depend on the topic. We highlight three main areas with evidence that universities might consider in more detail. First, in employment – as the slower improvement in senior authorship shows that women tend to end their academic career at the postdoctoral stage.^26^ There are institutional barriers, such as gender biases during recruitment, fewer opportunities for collaboration and inflexibility about accommodating career and family life.^27–29^ These seem to have been addressed – especially in psychology and psychiatry, with its increased and higher employment rates – and has been achieved through flexible working, mentoring, inclusive leadership and projects such as Athena SWAN and ADVANCE programmes.^30–33^

Second, the contribution of women may be less valued. In a recent study, women made up just under half the workforce (48.25%), but only 34.85% were authors.^34^ After contributing, they were either not named or not given priority in the author list.

The final barrier is the visibility of women’s research after publication, with women first or last authors in high-impact medical journals having far fewer citations than men. One explanation might be that men are more likely to self-cite.^35,36^ This needs further consideration for those investigating issues in mental health, as it might explain some of the variance in the poor rate of conversion from junior to senior positions.

### Gender equity research in the future – it is going to get more complicated

Our review stopped at the point where the COVID-19 pandemic started to have effects on publications, as it provided a potential disruption to the trajectories. This has been notable and was detrimental to women’s representation in authorship.^37–39^ This slowed pace is despite the media presence, at least in the UK, of notable women scientists such as those in the Oxford–AstraZeneca Vaccine Team (Sarah Gilbert, Catherine Green and Teresa Lambe), experts in airborne diseases like Cath Noakes, and the epidemiologist Devi Sridar. They are likely to be role models in the future. Similar role models in psychiatry and psychology need to be advanced, and perhaps the presence of women at the top of the Royal College of Psychiatrists will increase visibility in the media post-COVID-19.

Although classification methods had no effect on our estimates of women authorship, any future conversation about equality will soon require discussions of the additional challenge of intersectionality.^40^ How identities intersect (e.g. a Black woman or gay Muslim woman) can form unique barriers that are not simply additive.^41^ A growing body of research reflects this by demonstrating that many women’s experiences fail to be captured if issues of gender and race are analysed in separate silos.^42–44^ Understanding trends in intersectionality goes beyond the scope of our paper, but warrants future research, especially in mental health topics because it may be a potent factor in understanding and removing the barriers to women’s pursuit of an academic career.

### Strengths and limitations

We discovered many studies, mostly published post-1975, that examined the representation and trajectory of authorship for women academics. We followed PRISMA methods, checked our data quality via different sensitivity analyses, performed analyses to identify if any of our assumptions had affected the overall results and checked all potential confounders. This included consideration of the corpus of studies, interval length, interactions and removal of sampling periods before 1975. We are therefore confident that the overall results are robust and makes us confident in our baseline estimates. However, many publications did not provide sufficient information for a meta-analysis, especially the total sample size, which affected our ability to assess potential publication bias – although it seem unlikely that there was a bias. Finally, we assumed that last authors are typically senior authors, which is common in mental health research, but may differ in other disciplines.

In conclusion, this paper is unique in developing a robust baseline for the discipline of psychology and psychiatry, as we investigated potential methodological confounders. The story is half full or half empty, as mental health science performed better than other topics, but there was still a slow transition to senior authorship like other studies of STEMM publications. In the future, research investigating a growing trend upward or inertia for women in STEMM publications will need to update its methodologies if it is to prove useful in tackling existing social inequalities in academia. In terms of measurement, perhaps it is also time for academic journals to exercise humility, and reflect on their own processes of article selection.^45^

Our study shows that change continues to occur, but at a snail’s pace. It is time to be ambitious and set our sights on increasing the rate of change over the next 10 years, including initiatives for more visible role models of women. We have noted that this was achieved during the COVID-19 pandemic and, together with systemic changes within universities, could begin to accelerate the trajectories. But we know there is still a long way to go.

## Supporting information

Wykes et al. supplementary material 1 Wykes et al. supplementary material

Wykes et al. supplementary material 2 Wykes et al. supplementary material

## Data Availability

All data used for this publication are published in Supplementary Table 1, and so are publicly available. The analytical code is available on request.
