# Representation of women in scientific subjects: overview of systematic reviews investigating career progress in academic publishing with a focus on mental health – ERRATUM

**DOI:** 10.1192/bjo.2025.54

**Published:** 2025-04-07

**Authors:** Daniel Stahl, Ayse Kostem, Sanchita Garg, Emma Wilson-Lemoine, Til Wykes

**Keywords:** Career, gender, glass ceiling, sex, Science, Technology and Engineering, erratum

This article was originally published with the author list ordered wrongly as:

Til Wykes, Sanchita Garg, Daniel Stahl, Ayse Kostem and Emma Wilson-Lemoine.

The order of the author list should have been:

Daniel Stahl, Ayse Kostem, Sanchita Garg, Emma Wilson-Lemoine and Til Wykes.

This fault was introduced in the corrections process and the article has now been updated to reflect this change. The Publisher apologises for the error.
