# Supplementary material for: Representation of women in scientific subjects: overview of systematic reviews investigating career progress in academic publishing with a focus on mental health
Source: BJPsych Open. 2025 Mar 12;11(2):e49. doi: 10.1192/bjo.2024.820 (PMC12001945; doi:10.1192/bjo.2024.820)
Supplement: Wykes et al. supplementary material 1 — Wykes et al. supplementary material [file S2056472424008202sup001.docx]

**Supplementary Analyses**

**Figure 1s: Linear regression analyses using all available data**.

**Figure S1: Relationship between the time of data collection and proportion of a) women first authors and b) women last authors from 1910 to 2020.** The scatter plot shows the relative numbers of women for each study on the y-axis and the year collected on the x-axis. A quadratic trendline is fitted to the data.
