# Supplementary material for: Representation of women in scientific subjects: overview of systematic reviews investigating career progress in academic publishing with a focus on mental health
Source: BJPsych Open. 2025 Mar 12;11(2):e49. doi: 10.1192/bjo.2024.820 (PMC12001945; doi:10.1192/bjo.2024.820)
Supplement: Wykes et al. supplementary material 2 — Wykes et al. supplementary material [file S2056472424008202sup002.docx]

**Table S1**

The table provides the first author, year of publication, the title of study, Substudy (if applicable), Discipline of study, Discipline categorized in 7 categories, author classification method (using gender, sex or both), data extraction method (manual, algorithmic or both), Start and end year of reported proportions, total sample size and doi. In addition, we report if proportions are summarized per year or are averaged over a longer interval (Collapsed) if collapsed if intervals were longer than 5 years (Interval length) and if proportions were estimated from figures (Proportions estimated).

*West (2014) reports data from available publications from 1645 to 2011 but we only used data where categories were provided in figures.

**Table 1 Data used in all analyses**

| **Author** | **PubYear** | **Title** | **Discipline** | **Discipline Group** | **Author classify method** | **Extract method** | **Start Year** | **End Year** | **Total N** | **Doi** |
| --- | --- | --- | --- | --- | --- | --- | --- | --- | --- | --- |
| Ahmed | 2014 | Gender trends in radiation oncology in the United States: a 30-year analysis | Radiation Oncology | Medicine | Both | Manual | 1980 | 2012 | 1159 | 10.1016/j.ijrobp.201 |
| Amankwah | 2018 | Trends in Authorship Demographics for Manuscripts Published in The American Journal of Cardiology | Cardiology | Medicine | Gender | Algorithmic | 1958 | 2016 | 4329 | 10.1016/j.amjcard.20 |
| Amareskera | 2021 | Trends in Female Authorship in Cornea From 2007 to 2019. | Opthalmology | Medicine | Both | Manual | 2007 | 2018 | 2313 | 10.1097/ICO.00000000 |
| Amering | 2011 | The gender gap in high-impact psychiatry journals | Psychiatry | Psychology and Psychiatry | Gender | Manual | 1994 | 2007 | 950 | 10.1097/ACM.0b013e31 |
| Andersen | 2020 | COVID-19 medical papers have fewer women first authors than expected | COVID-19 Pandemic and Medicine | Medicine | Both | Algorithmic | 2019 | 2020 | 87266 | 10.7554/eLife.58807 |
| Andry | 2019 | An Analysis of Female Plastic Surgery Authorship: Where Are We Today? | Plastic Surgery | Medicine | Both |  | 2015 | 2015 | 2050 | 10.1097/PRS.00000000 |
| Arrighi-Allisan | 2019 | Gender trends in authorship of original otolaryngology publications: A fifteen-year perspective | Otolaryngology | Medicine | Gender | Manual | 2000 | 2015 | 9400 | 10.1002/lary.28372 |
| Aslan | 2020 | Women in Neurosurgery: Gender Differences in Authorship in High-Impact Neurosurgery Journals through the Last Two Decades | Neurosurgery | Medicine | Gender | Manual | 2003 | 2018 | 3247 | 10.1016/j.wneu.2020. |
| Ayyala | 2021 | Gender trends in authorship of Pediatric Radiology publications and impact of the COVID-19 pandemic. | Radiology | Medicine | Gender | Manual | 2017 | 2020 | 1018 | 10.1007/s00247-021-0 |
| Bagga | 2021 | Representation of Women as Authors of Rheumatology Research Articles | Rheumatology | Medicine | Gender | Manual | 2015 | 2019 | 7651 | 10.1002/art.41490 |
| Bailey | 2002 | Trends in author characteristics and diversity issues in the Journal of Marital and Family Therapy from 1990 to 2000 | Marital and Family Therapy | Psychology and Psychiatry | Gender | Manual | 1990 | 2000 | 283 | 10.1111/j.1752-0606. |
| Bendels | 2018 | Gender disparities in high-quality dermatology research: a descriptive bibliometric study on scientific authorships | Dermatology | Medicine | Both | Algorithmic | 2008 | 2017 | 23373 | 10.1136/bmjopen-2017 |
| Benjamens | 2020 | Gender Disparities in Authorships and Citations in Transplantation Research | Organ Transplantation | Medicine | Gender | Algorithmic | 1999 | 2018 | 15498 | 10.1097/TXD.00000000 |
| Bernardi | 2019 | Gender Disparity Among Surgical Peer-Reviewed Literature. | Surgery | Medicine | Both | Algorithmic | 2000 | 2017 | 560 | 10.1016/j.jss.2019.1 |
| Bernardi | 2019 | Gender Disparity in Authorship of Peer-Reviewed Medical Publications | Medical and Surgical | Medicine | Gender | Algorithmic | 2000 | 2017 | 1120 | 10.1016/j.amjms.2019 |
| Bhattacharyya, N | 2000 | Increased Female Authorship in Otolaryngology Over the Past Three Decades | Otolaryngology | Medicine | Gender | Manual | 1978 | 1998 | 2463 | 10.1097/00005537-200 |
| Brown | 2019 | Despite Growing Number of Women Surgeons, Authorship Gender Disparity in Orthopaedic Literature Persists Over 30 Years | Orthopaedics | Medicine | Gender | Manual | 1987 | 2017 | 1038 | 10.1097/CORR.0000000 |
| Brown | 2016 | Some Evidence for a Gender Gap in Personality and Social Psychology | Social Psychology | Psychology and Psychiatry | Gender | Manual | 2004 | 2013 | 563 | 10.1177/194855061664 |
| Brown | 2016 | Some Evidence for a Gender Gap in Personality and Social Psychology | Social Psychology | Psychology and Psychiatry | Gender | Manual | 2004 | 2013 | 531 | 10.1177/194855061664 |
| Burden | 2015 | Gender disparities in leadership and scholarly productivity of academic hospitalists | Medicine | Medicine | Both | Manual | 2006 | 2012 | 2172 | 10.1002/jhm.2340 |
| Campbell | 2018 | Collaboration Metrics Among Female and Male Researchers: A 5-Year Review of Publications in Major Radiology Journals. | Radiology | Medicine | Gender | Manual | 2011 | 2015 | 1934 | 10.1016/j.acra.2017. |
| Cannon | 2020 | Gender and Invited Authorship in the Journal of Pediatric Urology | Paediatric Urology | Medicine | Gender | Manual | 2006 | 2018 | 305 | 10.1016/j.urology.20 |
| Chary | 2021 | Gender disparity in critical care publications: a novel Female First Author Index | Critical Care Medicine | Medicine | Gender | Both | 2008 | 2018 | 7370 | 10.1186/s13613-021-0 |
| Chien | 2020 | Trends in Authorship of Original Scientific Articles in Journal of Glaucoma: An Analysis of 25 Years Since the Initiation of the Journal | Glaucoma | Medicine | Sex | Both | 1992 | 2017 | 642 | 10.1097/IJG.00000000 |
| Consky | 2020 | The Proportion of Female Authors in Oral and Maxillofacial Surgery Literature Has not Changed in 20 Years | Oral and Maxillofacial Surgery | Medicine | Gender | Manual | 1995 | 2015 | 983 | 10.1016/j.joms.2020. |
| Crockett | 2014 | The Career Development Quarterly: A 22-Year Publication Pattern Metastudy | Career Counselling | Psychology and Psychiatry | Gender | Manual | 1990 | 2011 | 642 | 10.1002/j.2161-0045. |
| da Silva | 2019 | The role of women in Brazilian ethnobiology: challenges and perspectives. | Ethnobiology | Natural Science | Both | Manual | 1989 | 2017 | 412 | 10.1186/s13002-019-0 |
| Dalal | 2020 | Mind the gap: Gendered publication trends in oncology | Oncology | Medicine | Both | Manual | 1990 | 2017 | 9189 | 10.1002/cncr.32818 |
| DeFilippis | 2021 | Gender Differences in Publication Authorship During COVID-19: A Bibliometric Analysis of High-Impact Cardiology Journals | Cardiology | Medicine | Gender | Algorithmic | 2019 | 2020 | 1627 | 10.1161/JAHA.120.019 |
| Dotson | 2011 | Women as authors in the pharmacy literature: 1989-2009 | Pharmacy | Medicine | Both | Manual | 1989 | 2009 | 648 | 10.2146/ajhp100597 |
| Dubey | 2016 | Trends in authorship based on gender and nationality in published neuroscience literature. | Neuroscience | Natural Science | Gender | Manual | 2002 | 2012 | 1084 | 10.4103/0028-3886.17 |
| Erden | 2015 | Longitudinal Analysis of Female Authorship of Psychiatry Articles in Turkey. | Psychiatry | Psychology and Psychiatry | Gender | Manual | 1980 | 2015 | 1961 | 10.5152/npa.2015.726 |
| Evans | 2011 | Reflecting on a Decade of Women's Publications in Four Top Political Science Journals | Political Science | Social Science | Both | Manual | 2000 | 2009 | 1880 | 10.1017/S10490965110 |
| Farooq | 2019 | Analysis of Authorship in Hepatopancreaticobiliary Surgery: Women Remain Underrepresented. | Hepatopancreaticobiliary Surgery | Medicine | Both | Manual | 2008 | 2018 | 1473 | 10.1007/s11605-019-0 |
| Fishman | 2017 | Gender Differences in the Authorship of Original Research in Pediatric Journals, 2001-2016. | Pediatrics | Medicine | Gender | Manual | 2001 | 2016 | 3895 | 10.1016/j.jpeds.2017 |
| Flexman | 2019 | Representation of female authors in the Canadian Journal of Anesthesia: a retrospective analysis of articles between 1954 and 2017 | Anesthesia | Medicine | Gender | Manual | 1954 | 2017 | 7370 | 10.1186/s13613-021-0 |
| Fuji | 2018 | Women among first authors in Japanese cardiovascular journal: An observational study | Cardiovascular medicine | Medicine | Gender | Manual | 2006 | 2015 | 7005 | 10.1007/s12630-019-0 |
| Gayet-Ageron | 2021 | Female authorship of covid-19 research in manuscripts submitted to 11 biomedical journals: cross sectional study | Biomedicine | Medicine | Gender | Algorithmic | 2018 | 2021 | 63259 | 10.1136/bmj.n2288 |
| Giuffrida | 2019 | Gender Imbalance in Authorship of Veterinary Literature: 1995 versus 2015 | Veterinary science | Medicine | Both | Manual | 1995 | 2015 | 2806 | 10.3138/jvme.1017-14 |
| Gu | 2017 | Progression of Authorship of Scientific Articles in The Journal of Hand Surgery, 1985-2015. | Hand Surgery | Medicine | Sex | Algorithmic | 1985 | 2015 | 892 | 10.1016/j.jhsa.2017. |
| Gupta | 2021 | Women Representation as First and Corresponding Authors in Neuroanesthesiology and Neurocritical Care Journals: A Retrospective Analysis | Neuroanesthesiology and Neurocritical Care | Medicine | Gender | Manual | 2015 | 2020 | 1164 | 10.1097/ANA.00000000 |
| Hagan | 2020 | Women Are Underrepresented and Receive Differential Outcomes at ASM Journals: a Six-Year Retrospective Analysis | Microbiology | Natural Science | Gender | Algorithmic | 2012 | 2018 | 79189 | 10.1128/mBio.01680-2 |
| Halderman | 2021 | Gender and authorship trends in rhinology, allergy, and skull-base literature from 2008 to 2018 | Rhinology, allergy, and skull-base literature | Medicine | Gender | Both | 2008 | 2018 | 2666 | 10.1002/alr.22793 |
| Hart | 2019 | Gender Trends in Authorship in Psychiatry Journals From 2008 to 2018. | Psychiatry | Psychology and Psychiatry | Gender | Algorithmic | 2008 | 2018 | 2529 | 10.1016/j.biopsych.2 |
| Haws | 2018 | Authorship Trends in Spine Publications From 2000 to 2015. | Spine medicine | Medicine | Sex | Manual | 2000 | 2015 | 2023 | 10.1097/BRS.00000000 |
| Hiller | 2019 | What Are the Rates and Trends of Women Authors in Three High-Impact Orthopaedic Journals from 2006-2017? | Orthopaedics | Medicine | Gender | Manual | 2006 | 2017 | 6292 | 10.1097/CORR.0000000 |
| Hsiehchen | 2019 | Prevalence of Female Authors in Case Reports Published in the Medical Literature | Medicine | Medicine | Sex | Manual | 2014 | 2015 | 20427 | 10.1001/jamanetworko |
| Hsu | 2021 | A comprehensive analysis of authorship trends in Skeletal Radiology since inception from 1976 to 2020 | Musculoskeletal radiology | Medicine | Gender | Algorithmic | 1976 | 2020 | 885 | 10.1007/s00256-021-0 |
| Jabbarpour | 2020 | Has Female Authorship in Family Medicine Research Evolved Over Time? | Family medicine | Medicine | Gender | Both | 2008 | 2017 | 2623 | 10.1370/afm.2584. |
| Jacobs | 2021 | Examining the Gender Gap in Emergency Medicine Research Publications. | Emergency medicine | Medicine | Gender | Both | 2013 | 2019 | 2980 | 10.1016/j.annemergme |
| Jagsi | 2006 | The "Gender Gap" in Authorship of Academic Medical Literature--A 35-Year Perspective. | Medicine | Medicine | Both | Manual | 1970 | 2004 | 7249 | 10.1056/NEJMsa053910 |
| Jarema | 1999 | Participation of women as authors and participants in articles published in the Journal of Organizational Behavior Management. | Behavioural Management | Psychology and Psychiatry | Both | Manual | 1977 | 1997 | 636 | 10.1080/01608061.201 |
| Keller | 2021 | Gender Differences in Authorship of Family Medicine Publications, 2002-2017 | Family medicine | Medicine | Gender | Manual | 2002 | 2017 | 1671 | 10.22454/FamMed.2021 |
| Kim | 2019 | A 46-year Analysis of Gender Trends in Academic Authorship in Orthopaedic Sports Medicine | Orthopaedic Sports | Medicine | Both | Algorithmic | 1972 | 2018 | 18354 | 10.5435/JAAOS-D-18-0 |
| Kongkiatkamon | 2010 | Gender disparities in prosthodontics: authorship and leadership, 13 years of observation | Prosthodontics | Medicine | Gender | Manual | 1995 | 2008 | 1202 | 10.1111/j.1532-849X. |
| Labinaz | 2019 | Female Authorship in Preclinical Cardiovascular Research: Temporal Trends and Influence on Experimental Design. | Cardiovascular medicine | Medicine | Sex | Algorithmic | 2006 | 2016 | 3396 | 10.1016/j.jacbts.201 |
| Lasnon | 2021 | Women Authors in Nuclear Medicine Journals: a Survey from 2014 to 2020. | Nuclear medicine | Medicine | Gender | Algorithmic | 2014 | 2020 | 12450 | 10.2967/jnumed.121.2 |
| Lennon | 2021 | Changes in the proportions of authors in Australian medical journals who were women, 2005-2018 | Medicine | Medicine | Gender | Algorithmic | 2005 | 2018 | 26972 | 10.5694/mja2.50998 |
| Lennox | 2019 | Patterns of North American Women Authorship in 2 Allergy/Immunology Journals: 1997-2017 | Allergy/Immunology | Medicine | Gender | Manual | 1997 | 2017 | 1374 | 10.1016/j.ja |
| Lerchenmuller | 2021 | Longitudinal analyses of gender differences in first authorship publications related to COVID-19 | COVID-19 | Medicine | Gender | Algorithmic | 2019 | 2020 | 483232 | 10.1136/bmjopen-2020 |
| Leung | 2021 | Gender differences in gastroenterology and hepatology authorship and editorial boards | Gastroenterology and hepatology | Medicine | Both | Manual | 2019 | 2019 | 8036 | 10.1016/j.gie.2021.0 |
| Liang | 2015 | Assessing the gap in female authorship in radiology: Trends over the past two decades. | Radiology | Medicine | Gender | Manual | 1993 | 2013 | 3786 | 10.1016/j.jacr.2015. |
| Long | 2015 | Female authorship in major academic gastroenterology journals: A look over 20 years | Gastroenterology | Medicine | Gender | Manual | 1992 | 2012 | 2730 | 10.1016/j.gie.2015.0 |
| Luc | 2021 | Trends in Female Authorship: A Bibliometric Analysis of The Annals of Thoracic Surgery | Thoraric Surgery | Medicine | Both | Both | 2013 | 2017 | 150 | 10.1016/j.athoracsur |
| Lydon | 2021 | Do Gender-Based Disparities in Authorship // Editorship Exist in Healthcare Simulation Journals? A Bibliometric Review of the Research | Healthcare Simulation | Multidisciplinary | Gender | Both | 2006 | 2018 | 873 | 10.1097/SIH.00000000 |
| Madden | 2021 | Gender in authorship and editorship in medical education journals: A bibliometric review | Medical Education | Medicine | Gender | Manual | 1970 | 2019 | 5749 | 10.1111/medu.14427 |
| Mahajan | 2020 | Does double-blind peer review impact gender authorship trends? An evaluation of two leading neurosurgical journals from 2010 to 2019. | Academic Neurosurgery | Medicine | Gender | Manual | 2010 | 2019 | 4028 | 10.3171/2020.6.JNS20 |
| Malchuk | 2021 | Gender Concordance of First and Senior Authors in Family Medicine Journals | Family Medicine | Medicine | Gender | Both | 2008 | 2017 | 2223 | 10.22454/FamMed.2021 |
| Mamtani | 2020 | Quantifying gender disparity in physician authorship among commentary articles in three high-impact medical journals: an observational study | Medicine | Medicine | Gender | Manual | 2014 | 2018 | 2087 | 10.1136/bmjopen-2019 |
| Manlove | 2018 | Authors and editors assort on gender and geography in high-rank ecological publications | Ecology | Natural Science | Gender | Algorithmic | 2015 | 2016 | 2351 | 10.1371/journal.pone |
| Mansour | 2012 | Five-Decade Profile of Women in Leadership Positions at Ophthalmic Publications | Ophthalmology | Medicine | Sex | Manual | 1969 | 2009 | 4034 | 10.1371/journal.pone |
| Marrone | 2020 | Does academic authorship reflect gender bias in pediatric surgery? An analysis of the Journal of Pediatric Surgery, 2007-2017 | Paediatric Surgery | Medicine | Gender | Both | 2007 | 2017 | 632 | 10.1016/j.jpedsurg.2 |
| Matchanova | 2020 | Gender disparities in the author bylines of articles published in clinical neuropsychology journals from 1985 to 2019 | Clinical Neuropsychology | Psychology and Psychiatry | Gender | Algorithmic | 1985 | 2019 | 10531 | 10.1080/13854046.202 |
| Matchanova | 2020 | Gender disparities in the author bylines of articles published in clinical neuropsychology journals from 1985 to 2019 | Clinical Neuropsychology | Psychology and Psychiatry | Gender | Algorithmic | 1985 | 2019 | 10531 | 10.1080/13854046.202 |
| Matchanova | 2020 | Gender disparities in the author bylines of articles published in clinical neuropsychology journals from 1985 to 2019 | Clinical Neuropsychology | Psychology and Psychiatry | Gender | Algorithmic | 1985 | 2019 | 10531 | 10.1080/13854046.202 |
| Mehta | 2021 | Trends in Published Palliative Care Research: A 15-Year Review | Palliative Care | Medicine | Gender | Manual | 2004 | 2018 | 4881 | 10.1177/104990912094 |
| Mendlowicz | 2011 | Is there a 'gender gap' in authorship of the main Brazilian psychiatric journals at the beginning of the 21st century? | Psychiatry | Psychology and Psychiatry | Both | Manual | 2001 | 2008 | 1036 | 10.1007/s11192-010-0 |
| Merriman | 2021 | The gender and geography of publishing: a review of sex/gender reporting and author representation in leading general medical and global health journals. | Medical and Global Health | Medicine | Both | Both | 2018 | 2019 | 542 | 10.1136/bmjgh-2021-0 |
| Miller | 2019 | Trends in Authorship in Anesthesiology Journals | Anesthesiology | Medicine | Gender | Manual | 2002 | 2017 | 2600 | 10.1213/ANE.00000000 |
| Molwitz | 2021 | Gender trends in authorships and publication impact in Academic Radiology-a 10-year perspective | Academic Radiology | Medicine | Both | Manual | 2007 | 2018 | 14360 | 10.1007/s00330-021-0 |
| Muric | 2021 | Gender Disparity in the Authorship of Biomedical Research Publications During the COVID-19 Pandemic: Retrospective Observational Study | Biomedical Research | Medicine | Gender | Algorithmic | 2019 | 2020 | 78950 | 10.2196/25379 |
| Nguyen | 2021 | Impact of COVID-19 on longitudinal ophthalmology authorship gender trends | Ophthalmology | Medicine | Gender | Both | 2019 | 2020 | 528 | 10.1007/s00417-021-0 |
| Nguyen | 2021 | Gender Gap in Neurology Research Authorship (1946-2020) | Neurology | Medicine | Gender | Both | 1946 | 2020 | 299843 | 10.3389/fneur.2021.7 |
| Nguyen | 2021 | Women as first authors in key pharmacy journals: Analysis by publication type | Pharmacy | Medicine | Gender | Algorithmic | 2007 | 2017 | 14658 | 10.1016/j.japh.2020. |
| Ouyang | 2018 | Sex Disparities in Authorship Order of Cardiology Scientific Publications. | Cardiology | Medicine | Sex | Algorithmic | 1980 | 2017 | 72362 | 10.1161/CIRCOUTCOMES |
| Pagel | 2019 | Gender Differences in Authorship in the Journal of Cardiothoracic and Vascular Anesthesia: A 28-Year Analysis of Publications Originating From the United States, 1990-2017 | Anesthesiology | Medicine | Both | Manual | 1990 | 2017 | 1195 | 10.1053/j.jvca.2018. |
| Pagel | 2019 | A 50-year analysis of gender differences in United States authorship of original research articles in two major anesthesiology journals | Anesthesiology | Medicine | Gender | Manual | 1967 | 2017 | 1350 | 10.1007/s11192-019-0 |
| Pasick | 2020 | Sex Bias in Laryngology Research and Publishing. | Laryngology | Medicine | Both | Both | 2019 | 2019 | 1340 | 10.1016/j.jvoice.20 |
| Pastor-Cabeza | 2021 | Women's role in neurosurgical research: is the gender gap improving? | Neurosurgical research | Medicine | Both | Manual | 2009 | 2019 | 1328 | 10.3171/2020.12.FOCU |
| Pebdani | 2018 | Examining Gender Differences in Rehabilitation Counseling Publication: 1990-2015 | Counseling | Psychology and Psychiatry | Gender | Manual | 1990 | 2015 | 4074 | 10.1891/2168-6653.32 |
| Pico | 2020 | First Authorship Gender Gap in the Geosciences | Geosciences | Natural Science | Gender | Algorithmic | 2013 | 2019 | 35183 | 10.1029/2020EA001203 |
| Piper | 2016 | Gender Trends in Radiology Authorship: A 35-Year Analysis | Radiology | Medicine | Gender | Manual | 1978 | 2013 | 4217 | 10.2214/AJR.15.15116 |
| Polanco | 2020 | Gender Differences in Hepatology Medical Literature | Hepatology | Medicine | Gender | Manual | 2014 | 2016 | 4083 | 10.1007/s10620-019-0 |
| Prior | 2021 | Analysis of Author Gender in the Pediatric Orthopaedic Literature from 2011 to 2020 | Pediatric Orthopaedics | Medicine | Gender | Both | 2011 | 2020 | 4097 | 10.1097/BPO.00000000 |
| Purdy | 2021 | Gender differences in publication in emergency medicine journals | Emergency medicine | Medicine | Gender | Algorithmic | 2015 | 2018 | 4166 | 10.1016/j.ajem.2021. |
| Pyatigorskaya | 2017 | Women authorship in radiology research in France: An analysis of the last three decades | Radiology | Medicine | Gender | Manual | 1984 | 2014 | 1189 | 10.1016/j.diii.2017. |
| Qureshi | 2020 | Authorship diversity among systematic reviews in eyes and vision | Eyes and Vision | Medicine | Gender | Both | 2007 | 2018 | 626 | 10.1186/s13643-020-0 |
| Ravi | 2021 | Systematic analysis of authorship demographics in global surgery. | Global Surgery | Medicine | Gender | Both | 2016 | 2020 | 1240 | 10.1136/bmjgh-2021-0 |
| Rickard | 2020 | Female Authorship Publishing Trends and Forecasting in Pediatric Urology: Are We Closer to Gender Equality? | Pediatric Urology | Medicine | Gender | Manual | 1990 | 2019 | 750 | 10.1016/j.urology.20 |
| Russell | 2019 | A Bibliometric Study of Authorship and Collaboration Trends Over the Past 30 Years in Four Major Musculoskeletal Science Journals | Musculoskeletal Science | Medicine | Gender | Algorithmic | 1985 | 2015 | 2776 | 10.1007/s00223-018-0 |
| Rynecki | 2019 | How Well Represented Are Women Orthopaedic Surgeons and Residents on Major Orthopaedic Editorial Boards and Publications? | Orthopaedics | Medicine | Gender | Manual | 1997 | 2017 | 8753 | 10.1097/CORR.0000000 |
| Salinaro | 2018 | Gender trends in gynecologic oncology authorship: Implications for the critical evaluation of gender distribution in academic rank and leadership positions | Gynecologic Oncology | Medicine | Gender | Manual | 2000 | 2015 | 2039 | 10.1016/j.ygyno.2018 |
| Schauwecker | 2021 | Gender Prevalence and Trends in Otology and Neurotology Publications | Otology and Neurotology | Medicine | Gender | Manual | 2000 | 2019 | 4411 | 10.1097/MAO.00000000 |
| Schrager | 2011 | Gender and First Authorship of Papers in Family Medicine Journals 2006-2008. | Family medicine | Medicine | Gender | Manual | 2006 | 2008 | 2126 | http://www.ncbi.nlm. |
| Sela | 2021 | Gender Differences in Authorship Among Transplant Physicians: Are We Bridging the Gap? | Transplantation | Medicine | Gender | Manual | 2008 | 2017 | 2530 | 10.1016/j.jss.2020.0 |
| Shah | 2021 | Gender parity in scientific authorship in a National Institute for Health Research Biomedical Research Centre: a bibliometric analysis | Biomedical Research | Medicine | Gender | Both | 2012 | 2017 | 2409 | 10.1136/bmjopen-2020 |
| Shah | 2013 | Trends in female representation in published ophthalmology literature, 2000-2009 | Ophthalmology | Medicine | Both | Manual | 2000 | 2009 | 17271 | 10.5693/djo.01.2013. |
| Sheridan | 2018 | A bibliometric analysis assessing temporal changes in publication and authorship characteristics in The Knee from 1996 to 2016 | Medicine | Medicine | Gender |  | 1996 | 2016 | 413 | 10.1016/j.knee.2018. |
| Shukla | 2021 | Does Gender Matter in Academic Surgery? Author and Mentor Gender Impact Publication Citations in Surgical Research. | Surgical Research | Medicine | Gender | Both | 2000 | 2019 | 5787 | 10.1016/j.urology.20 |
| Sidhu | 2009 | The gender imbalance in academic medicine: a study of female authorship in the United Kingdom | Medicine | Medicine | Both | Manual | 1970 | 2004 | 6468 | 10.1258/jrsm.2009.08 |
| Sing | 2017 | Gender trends in authorship of spine-related academic literature-a 39-year perspective | Spine medicine | Medicine | Both | Algorithmic | 1978 | 2016 | 33480 | 10.1016/j.spinee.201 |
| Skinner | 1999 | Female publication patterns in School Psychology Review, Journal of School Psychology, and School Psychology Quarterly from 1985-1994. | Psychology | Psychology and Psychiatry | Gender | Manual | 1985 | 1994 | 1616 | 10.1080/02796015.199 |
| Sotudeh | 2018 | Gender differences in scientific productivity and visibility in core neurosurgery journals: Citations and social media metrics | Neurosurgery | Medicine | Both | Manual | 2012 | 2014 | 11127 | 10.1093/reseval/rvy0 |
| Stinson | 2003 | Participation of women in human biology, 1975-2001 | Biology | Natural Science | Both | Manual | 1975 | 2001 | 1616 | 10.1002/ajhb.10160 |
| Strand | 2018 | Trends in female authorship in research papers on eating disorders: 20-year bibliometric study. | Psychiatry | Psychology and Psychiatry | Gender | Manual | 1997 | 2016 | 5429 | 10.1192/bjo.2017.8 |
| Strand | 2018 | Trends in female authorship in research papers on eating disorders: 20-year bibliometric study. | Psychiatry | Psychology and Psychiatry | Gender | Manual | 1997 | 2016 | 5429 | 10.1192/bjo.2017.8 |
| Strand | 2018 | Trends in female authorship in research papers on eating disorders: 20-year bibliometric study. | Psychiatry | Psychology and Psychiatry | Gender | Manual | 1997 | 2016 | 5429 | 10.1192/bjo.2017.8 |
| Subramaniam | 2021 | Equal Opportunity: Women Representation on Editorial Boards and Authorship of Editorials in Gastroenterology and Hepatology Journals | Gastroenterology and Hepatology | Medicine | Both | Both | 1985 | 2020 | 1705 | 10.14309/ajg.0000000 |
| Süßenbacher | 2017 | Gender-gaps and glass ceilings: A survey of gender-specific publication trends in Psychiatry between 1994 and 2014 | Psychiatry | Psychology and Psychiatry | Gender | Manual | 1994 | 2014 | 10347 | 10.1016/j.eurpsy.201 |
| Swarnkar | 2021 | Women in Cosmetic Plastic Surgery: An Analysis of Female Authorship in Cosmetic Plastic Surgery Over the Last 10 Years | Cosmetic Plastic Surgery | Medicine | Gender | Manual | 2008 | 2019 | 4358 | 10.1177/074880682199 |
| Taha | 2021 | Increases in female academic productivity and female mentorship highlight sustained progress in previously identified neurosurgical gender disparities | Neurosurgery | Medicine | Gender | Algorithmic | 2002 | 2019 | 66546 | 10.3171/2020.12.FOCU |
| Takayanagui | 2009 | The increasing female participation in authorship of articles published in neurology in Brazil | Neurology | Medicine | Both |  | 1945 | 2005 | 35 | 10.1590/S0004-282X20 |
| Terry | 1996 | Authorship in College & Research Libraries revisited: Gender, institutional affiliation, collaboration. | Library and Information Sciences | Information Science | Both |  | 1939 | 1994 | 381 | 10.5860/CRL_57_04_37 |
| Thomson | 2019 | Visibility and representation of women in multiple sclerosis research. | Multiple Sclerosis | Medicine | Gender | Manual | 2017 | 2017 | 2532 | 10.1212/WNL.00000000 |
| Van Doren | 2019 | Sex differences in publication volume and quality in congenital heart disease: Are women disadvantaged? | Congenital Heart Disease | Medicine | Both | Algorithmic | 2006 | 2015 | 35118 | 10.1136/openhrt-2018 |
| Vasti | 2021 | Gender Disparities in Cardiology-Related COVID-19 Publications | Cardiology and COVID-19 | Medicine | Both | Algorithmic | 2020 | 2020 | 841 | 10.1007/s40119-021-0 |
| Vela | 2012 | Participation of women in software engineering publications | Software Engineering | Engineering | Gender | Manual | 2007 | 2008 | 1266 | 10.1007/s11192-012-0 |
| Vranas | 2020 | Gender Differences in Authorship of Critical Care Literature | Critical Care | Medicine | Gender | Both | 2008 | 2018 | 18483 | 10.1164/rccm.201910- |
| Webb | 2021 | First and last authorship by gender in emergency medicine publications- a comparison of 2008 vs. 2018 | Emergency Medicine | Medicine | Gender | Manual | 2008 | 2018 | 848 | 10.1016/j.ajem.2020. |
| West | 2013 | The Role of Gender in Scholarly Authorship | Natural sciences, Social sciences, and Humanities | Multidisciplinary | Gender | Algorithmic | 1665 | 2011 | ###### | 10.1371/journal.pone |
| Whitley | 2021 | Gender Differences in Authorship in Urology: a Five-year Review of Publications in Five High-impact Journals | Urology | Medicine | Gender | Manual | 2014 | 2019 | 8653 | 10.1016/j.urology.20 |
| Whitley | 2021 | Gender Differences in Authorship in Urology: a Five-year Review of Publications in Five High-impact Journals | Urology | Medicine | Gender | Manual | 2014 | 2019 | 8653 | 10.1016/j.urology.20 |
| Whitley | 2021 | Gender Differences in Authorship in Urology: a Five-year Review of Publications in Five High-impact Journals | Urology | Medicine | Gender | Manual | 2014 | 2019 | 8653 | 10.1016/j.urology.20 |
| Whitley | 2021 | Gender Differences in Authorship in Urology: a Five-year Review of Publications in Five High-impact Journals | Urology | Medicine | Gender | Manual | 2014 | 2019 | 8653 | 10.1016/j.urology.20 |
| Whitley | 2021 | Gender Differences in Authorship in Urology: a Five-year Review of Publications in Five High-impact Journals | Urology | Medicine | Gender | Manual | 2014 | 2019 | 8653 | 10.1016/j.urology.20 |
| Williams | 2018 | Mirror on the Field: Gender, Authorship, and Research Methods in Higher Education's Leading Journals | Higher Education | Social Science | Gender | Manual | 2006 | 2010 | 406 | 10.1080/00221546.201 |
| Wilson | 1998 | Patterns in publishing in three North American herpetological journals: Gender biases. | Herpetology | Medicine | Both | Manual | 1973 | 1993 | 97313 | 10.1655/0018-0831-77 |
| Xiao | 2018 | Characterizing the Impact of Women in Academic IR: A 12-Year Analysis | Interventional Radiology | Medicine | Both | Algorithmic | 2006 | 2017 | 3017 | 10.1016/j.jvir.2018. |
| Xu | 2020 | Trends in Gender Disparities in Authorship of Arthroplasty Research | Arthoplasty | Medicine | Both | Algorithmic | 2002 | 2019 | 14692 | 10.2106/JBJS.20.0025 |
| Yalamanchali | 2021 | Trends in Female Authorship in Major Journals of 3 Oncology Disciplines, 2002-2018 | Oncology | Medicine | Gender | Algorithmic | 2002 | 2018 | 58368 | 10.1001/jamanetworko |
| Yalamanchali | 2021 | Trends in Female Authorship in Major Journals of 3 Oncology Disciplines, 2002-2018 | Oncology | Medicine | Gender | Algorithmic | 2002 | 2018 | 58368 | 10.1001/jamanetworko |
| Yalamanchali | 2021 | Trends in Female Authorship in Major Journals of 3 Oncology Disciplines, 2002-2018 | Oncology | Medicine | Gender | Algorithmic | 2002 | 2018 | 58368 | 10.1001/jamanetworko |
| Yalamanchali | 2021 | Trends in Female Authorship in Major Journals of 3 Oncology Disciplines, 2002-2018 | Oncology | Medicine | Gender | Algorithmic | 2002 | 2018 | 58368 | 10.1001/jamanetworko |
| Yalamanchali | 2021 | Trends in Female Authorship in Major Journals of 3 Oncology Disciplines, 2002-2018 | Oncology | Medicine | Gender | Algorithmic | 2002 | 2018 | 58368 | 10.1001/jamanetworko |
| Yalamanchali | 2021 | Trends in Female Authorship in Major Journals of 3 Oncology Disciplines, 2002-2018 | Oncology | Medicine | Gender | Algorithmic | 2002 | 2018 | 58368 | 10.1001/jamanetworko |
| Yuan | 2010 | Gender trends in dental leadership and academics: a twenty-two-year observation | Dentistry | Medicine | Gender | Manual | 1986 | 2008 | 5773 | 10.1002/j.0022-0337. |
| Yun | 2015 | Closing the gender gap: Increased female authorship in AJR and radiology. | Radiology | Medicine | Gender | Manual | 1991 | 2013 | 5523 | 10.2214/AJR.14.14225 |
| Zehetbauer | 2021 | Gender-specific analysis of the authors and the editorial board of Naunyn-Schmiedeberg's Archives of Pharmacology from 2000 to 2020 | Pharmacology | Natural Science | Gender | Both | 2000 | 2020 | 651 | 10.1007/s00210-021-0 |

**References of studies used for this publication**

1. Ahmed, A. A., Egleston, B., Holliday, E., Eastwick, G., Takita, C., & Jagsi, R. (2014). Gender trends in radiation oncology in the United States: a 30-year analysis. *Int J Radiat Oncol Biol Phys*, *88*(1), 33-38. <https://doi.org/10.1016/j.ijrobp.2013.09.025>
2. Amankwah, N., Park, M., Gu, A., & Choi, B. G. (2018). Trends in Authorship Demographics for Manuscripts Published in The American Journal of Cardiology. *Am J Cardiol*, *122*(7), 1255-1259. <https://doi.org/10.1016/j.amjcard.2018.06.026>
3. Amarasekera, D. C., Lam, S. S., Rapuano, C. J., & Syed, Z. A. (2021). Trends in Female Authorship in Cornea From 2007 to 2019. *Cornea*, *40*(9). <https://doi.org/10.1097/ICO.0000000000002598>
4. Amering, M., Schrank, B., & Sibitz, I. (2011). The gender gap in high-impact psychiatry journals. *Acad Med*, *86*(8), 946-952. <https://doi.org/10.1097/ACM.0b013e3182222887>
5. Andersen, J. P., Nielsen, M. W., Simone, N. L., Lewiss, R. E., & Jagsi, R. (2020). COVID-19 medical papers have fewer women first authors than expected [Comparative Study]. *eLife*, *9*(06), 15. <https://doi.org/10.7554/eLife.58807>
6. Arrighi‐Allisan, A. E., Shukla, D. C., Meyer, A. M., Kidwai, S. M., Barazani, S. H., Cosetti, M. K., Teng, M. S., & Arrighi-Allisan, A. E. (2020). Gender Trends in Authorship of Original Otolaryngology Publications: A Fifteen-Year Perspective. *Laryngoscope*, *130*(9), 2126-2132. <https://doi.org/10.1002/lary.28372>
7. Aslan, A., Kuzucu, P., Karaaslan, B., & Borcek, A. O. (2020). Women in Neurosurgery: Gender Differences in Authorship in High-Impact Neurosurgery Journals through the Last Two Decades. *World Neurosurgery*, *138*, 374-380.
8. Ayyala, R. S., & Trout, A. T. (2021). Gender trends in authorship of Pediatric Radiology publications and impact of the COVID-19 pandemic. *Pediatric radiology*. <https://doi.org/10.1007/s00247-021-05213-6>
9. Bagga, E., Stewart, S., Gamble, G. D., Hill, J., Grey, A., & Dalbeth, N. (2021). Representation of Women as Authors of Rheumatology Research Articles. *Arthritis & Rheumatology*, *73*(1), 162-167. <https://doi.org/10.1002/art.41490>
10. Bailey, C. E., Pryce, J., & Walsh, F. (2002). Trends in author characteristics and diversity issues in the Journal of Marital and Family Therapy from 1990 to 2000. *J Marital Fam Ther*, *28*(4), 479-486. <https://doi.org/10.1111/j.1752-0606.2002.tb00372.x>
11. Bendels, M. H. K., Dietz, M. C., Bruggmann, D., Oremek, G. M., Schoffel, N., & Groneberg, D. A. (2018). Gender disparities in high-quality dermatology research: a descriptive bibliometric study on scientific authorships. *BMJ Open*, *8*(4), e020089. <https://doi.org/10.1136/bmjopen-2017-020089>
12. Benjamens, S., Banning, L. B. D., van den Berg, T. A. J., & Pol, R. A. (2020). Gender Disparities in Authorships and Citations in Transplantation Research. *Transplantation direct*, *6*(11). <https://doi.org/10.1097/TXD.0000000000001072>
13. Bernard, C., Pommier, R., Vilgrain, V., & Ronot, M. (2020). Gender gap in articles published in European Radiology and CardioVascular and Interventional Radiology: evolution between 2002 and 2016. *European Radiology*, *30*(2), 1011-1019.
14. Bernardi, K., Lyons, N. B., Huang, L., Holihan, J. L., Olavarria, O. A., Loor, M. M., Ko, T. C., & Liang, M. K. (2020). Gender Disparity Among Surgical Peer-Reviewed Literature. *J Surg Res*, *248*, 117-122. <https://doi.org/10.1016/j.jss.2019.11.007>
15. Bernardi, K., Lyons, N. B., Huang, L., Holihan, J. L., Olavarria, O. A., Martin, A. C., Milton, A. N., Loor, M. M., Zheng, F., Tyson, J. E., Ko, T. C., & Liang, M. K. (2020). Gender Disparity in Authorship of Peer-Reviewed Medical Publications. *AMERICAN JOURNAL OF THE MEDICAL SCIENCES*, *360*(5). <https://doi.org/10.1016/j.amjms.2019.11.005>
16. Bhattacharyya, N., & Shapiro, N. L. (2000). Increased female authorship in otolaryngology over the past three decades. *Laryngoscope*, *110*(3 Pt 1), 358-361. <https://doi.org/10.1097/00005537-200003000-00005>
17. Bonham, K. S., & Stefan, M. I. (2017). Women are underrepresented in computational biology: An analysis of the scholarly literature in biology, computer science and computational biology. *PLoS Comput Biol*, *13*(10), e1005134. <https://doi.org/10.1371/journal.pcbi.1005134>
18. Brown, A. J., & Goh, J. X. (2016). Some Evidence for a Gender Gap in Personality and Social Psychology [Article]. *Social Psychological and Personality Science*, *7*(5), 437-443. <https://doi.org/10.1177/1948550616644297>
19. Brown, M. A., Erdman, M. K., Munger, A. M., & Miller, A. N. (2020). Despite Growing Number of Women Surgeons, Authorship Gender Disparity in Orthopaedic Literature Persists Over 30 Years. *Clinical Orthopaedics & Related Research®*, *478*(7), 1542-1552. <https://doi.org/10.1097/CORR.0000000000000849>
20. Burden, M., Frank, M. G., Keniston, A., Chadaga, S. R., Czernik, Z., Echaniz, M., Griffith, J., Mintzer, D., Munoa, A., Spence, J., Statland, B., Teixeira, J. P., Zoucha, J., Lones, J., & Albert, R. K. (2015). Gender disparities in leadership and scholarly productivity of academic hospitalists. *J Hosp Med*, *10*(8), 481-485. <https://doi.org/10.1002/jhm.2340>
21. Campbell, J. C., Yoon, S. C., & Grimm, L. J. (2018). Collaboration Metrics Among Female and Male Researchers: A 5-Year Review of Publications in Major Radiology Journals. *Acad Radiol*, *25*(7), 951-954. <https://doi.org/10.1016/j.acra.2017.12.034>
22. Campbell, J. C., Yoon, S. C., & Grimm, L. J. (2019). Authorship and Impact of Gender-Specific Research in Major Radiology Journals. *J Am Coll Radiol*, *16*(2), 240-243. <https://doi.org/10.1016/j.jacr.2018.08.024>
23. Cannon, S., Ahn, J., Shnorhavorian, M., Kieran, K., & Merguerian, P. (2020). Gender and Invited Authorship in the Journal of Pediatric Urology. *Urology*, *145*, 211-215. <https://doi.org/10.1016/j.urology.2020.05.097>
24. Chary, S., Amrein, K., Soeteman, D. I., Mehta, S., & Christopher, K. B. (2021). Gender disparity in critical care publications: a novel Female First Author Index. *Annals of Intensive Care*. <http://dx.doi.org/10.1186/s13613-021-00889-3>
25. Chien, J. L., Wu, B. P., Nayer, Z., Grits, D., Rodriguez, G., Gu, A., Ghassibi, M. P., Chien, G. F., Oliveira, C., Stamper, R. L., Van Tassel, S. H., Muylaert, S., & Belyea, D. A. (2020). Trends in Authorship of Original Scientific Articles in Journal of Glaucoma: An Analysis of 25 Years Since the Initiation of the Journal. *Journal of Glaucoma*, *29*(7), 561-566. <https://doi.org/10.1097/IJG.0000000000001503>
26. Consky, E. K., Bradshaw, S. M., Wein, A. N., & Abramowicz, S. (2020). The Proportion of Female Authors in Oral and Maxillofacial Surgery Literature Has not Changed in 20 Years. *Journal of Oral & Maxillofacial Surgery (02782391)*, *78*(6), 877-881. <https://doi.org/10.1016/j.joms.2020.03.011>
27. da Silva, T. C., de Medeiros, P. M., Hanazaki, N., da Fonseca-Kruel, V. S., Hora, J. S. L., & de Medeiros, S. G. (2019). The role of women in Brazilian ethnobiology: challenges and perspectives. *J Ethnobiol Ethnomed*, *15*(1), 44. <https://doi.org/10.1186/s13002-019-0322-3>
28. Dalal, N. H., Chino, F., Williamson, H., Beasley, G. M., Salama, A. K. S., & Palta, M. (2020). Mind the gap: Gendered publication trends in oncology. *Cancer (0008543X)*, *126*(12), 2859-2865. <https://doi.org/10.1002/cncr.32818>
29. DeFilippis, E. M., Sinnenberg, L., Mahmud, N., Wood, M. J., Hayes, S. N., Michos, E. D., & Reza, N. (2021). Gender Differences in Publication Authorship During COVID-19: A Bibliometric Analysis of High-Impact Cardiology Journals. *Journal of the American Heart Association*, *10*(5), 1-6. <https://doi.org/10.1161/JAHA.120.019005>
30. Dotson, B. (2011). Women as authors in the pharmacy literature: 1989-2009. *Am J Health Syst Pharm*, *68*(18), 1736-1739. <https://doi.org/10.2146/ajhp100597>
31. Dubey, D., Sawhney, A., Atluru, A., Amritphale, A., Dubey, A., & Trivedi, J. (2016). Trends in authorship based on gender and nationality in published neuroscience literature. *Neurol India*, *64*(1), 97-100. <https://doi.org/10.4103/0028-3886.173643>
32. Erden Aki, O., Ozcelik Eroglu, E., & Uslu, E. (2015). Longitudinal Analysis of Female Authorship of Psychiatry Articles in Turkey. *Noro Psikiyatr Ars*, *52*(1), 95-98. <https://doi.org/10.5152/npa.2015.7265>
33. Evans, H. K., & Moulder, A. (2011). Reflecting on a Decade of Women's Publications in Four Top Political Science Journals [Article]. *Ps-Political Science & Politics*, *44*(4), 793-798. <https://doi.org/10.1017/s1049096511001296>
34. Farooq, A., Sahara, K., Muneeb, A., Farooq, K., Tsilimigras, D. I., Merath, K., Mehta, R., Paredes, A., Wu, L., Hyer, J. M., Beal, E., Pawlik, T. M., & Dillhoff, M. E. (2020). Analysis of Authorship in Hepatopancreaticobiliary Surgery: Women Remain Underrepresented. *Journal of Gastrointestinal Surgery*, *24*(9), 2070-2076. <https://doi.org/10.1007/s11605-019-04340-8>
35. Flexman, A. M., Parmar, A., & Lorello, G. R. (2019). Representation of female authors in the Canadian Journal of Anesthesia: a retrospective analysis of articles between 1954 and 2017. *Can J Anaesth*, *66*(5), 495-502. <https://doi.org/10.1007/s12630-019-01328-5> (Representation des femmes auteures dans le Journal canadien d'anesthesie : analyse retrospective des articles parus de 1954 a 2017.)
36. Fujii, T., Matsuyama, T., Takeuchi, J., Hara, M., Kitamura, T., & Yamauchi-Takihara, K. (2018). Women among First Authors in Japanese Cardiovascular Journal An Observational Study [Article]. *International Heart Journal*, *59*(2), 372-377. <https://doi.org/10.1536/ihj.17-187>
37. Gayet-Ageron, A., Ben Messaoud, K., Richards, M., & Schroter, S. (2021). Female authorship of covid-19 research in manuscripts submitted to 11 biomedical journals: cross sectional study [Research Support, Non-U.S. Gov't]. *BMJ*, *375*, n2288.
38. Giuffrida, M. A., Burton, J. H., Dechant, J. E., & Winter, A. (2019). Gender Imbalance in Authorship of Veterinary Literature: 1995 versus 2015. *J Vet Med Educ*, *46*(4), 429-437. <https://doi.org/10.3138/jvme.1017-141r>
39. Gu, A., Almeida, N., Cohen, J. S., Peck, K. M., & Merrell, G. A. (2017). Progression of Authorship of Scientific Articles in The Journal of Hand Surgery, 1985-2015. *J Hand Surg Am*, *42*(4), 291 e291-291 e296. <https://doi.org/10.1016/j.jhsa.2017.01.005>
40. Gupta, N., Banerjee, S., Choudhury, K. J., & Prabhakar, H. (2021). Women Representation as First and Corresponding Authors in Neuroanesthesiology and Neurocritical Care Journals: A Retrospective Analysis. *JOURNAL OF NEUROSURGICAL ANESTHESIOLOGY*, *33*(4). <https://doi.org/10.1097/ANA.0000000000000788>
41. Hagan, A. K., Topcuoglu, B. D., Gregory, M. E., Barton, H. A., & Schloss, P. D. (2020). Women Are Underrepresented and Receive Differential Outcomes at ASM Journals: a Six-Year Retrospective Analysis [Research Support, Non-U.S. Gov't]. *mBio*, *11*(6), 01.
42. Halderman, A. A., Rao, A., Desai-Markowski, S., Yang, A., Luong, A. U., O'Brien, E., Gray, S. T., Lal, D., Lin, S. Y., Orlandi, R., & Wise, S. K. (2021). Gender and authorship trends in rhinology, allergy, and skull-base literature from 2008 to 2018. *INTERNATIONAL FORUM OF ALLERGY & RHINOLOGY*, *11*(9). <https://doi.org/10.1002/alr.22793>
43. Hart, K. L., Frangou, S., & Perlis, R. H. (2019). Gender Trends in Authorship in Psychiatry Journals From 2008 to 2018. *Biol Psychiatry*, *86*(8), 639-646. <https://doi.org/10.1016/j.biopsych.2019.02.010>
44. Haws, B. E., Khechen, B., Movassaghi, K., Yom, K. H., Guntin, J. A., Cardinal, K. L., Shoshana, N. B., & Singh, K. (2018). Authorship Trends in Spine Publications From 2000 to 2015. *Spine (Phila Pa 1976)*, *43*(17), 1225-1230. <https://doi.org/10.1097/BRS.0000000000002585>
45. Hiller, K. P., Boulos, A., Tran, M. M., & Cruz, A. I., Jr. (2020). What Are the Rates and Trends of Women Authors in Three High-impact Orthopaedic Journals from 2006-2017? [Comparative Study]. *Clinical Orthopaedics & Related Research*, *478*(7), 1553-1560.
46. Hoogman, M., Onnink, M., Cools, R., Aarts, E., Kan, C., Arias Vasquez, A., Buitelaar, J., & Franke, B. (2013). The dopamine transporter haplotype and reward-related striatal responses in adult ADHD. *Eur Neuropsychopharmacol*, *23*(6), 469-478. <https://doi.org/10.1016/j.euroneuro.2012.05.011>
47. Hsiehchen, D., Hsieh, A., & Espinoza, M. (2019). Prevalence of Female Authors in Case Reports Published in the Medical Literature. *JAMA Netw Open*, *2*(5), e195000. <https://doi.org/10.1001/jamanetworkopen.2019.5000>
48. Hsu, A. L., Konner, M., Muttreja, A., Lee, C.-H., Chien, J. L., & Irish, R. D. (2021). A comprehensive analysis of authorship trends in Skeletal Radiology since inception from 1976 to 2020. *SKELETAL RADIOLOGY*. <https://doi.org/10.1007/s00256-021-03810-y>
49. Jabbarpour, Y., Wilkinson, E., Coffman, M., & Mieses, A. (2020). Has Female Authorship in Family Medicine Research Evolved Over Time? *Annals of Family Medicine*, *18*(6), 496-502. <https://doi.org/10.1370/afm.2584>
50. Jacobs, S. A., Van Loveren, K., Gottlieb, D., Brave, M., Loman, J., Weinman, L., & Kwon, N. (2021). Examining the Gender Gap in Emergency Medicine Research Publications. *Annals of emergency medicine*. <https://doi.org/10.1016/j.annemergmed.2021.08.008>
51. Jagsi, R., Guancial, E. A., Worobey, C. C., Henault, L. E., Chang, Y., Starr, R., Tarbell, N. J., & Hylek, E. M. (2006). The "gender gap" in authorship of academic medical literature--a 35-year perspective. *N Engl J Med*, *355*(3), 281-287. <https://doi.org/10.1056/NEJMsa053910>
52. Jorge, A., Bolster, M., Fu, X., Blumenthal, D. M., Gross, N., Blumenthal, K. G., & Wallace, Z. (2021). The Association Between Physician Gender and Career Advancement Among Academic Rheumatologists in the United States. *Arthritis & Rheumatology*, *73*(1), 168-172. <https://doi.org/10.1002/art.41492>
53. Keller, T., Wilson, M., Chung, K., Andrilla, C. H., Evans, D., Cawse-Lucas, J., Andrilla, C. H. A., & Evans, D. V. (2021). Gender Differences in Authorship of Family Medicine Publications, 2002-2017. *Family Medicine*, *53*(6), 416-422. <https://doi.org/10.22454/FamMed.2021.866524>
54. Kim, C. Y., Sivasundaram, L., Trivedi, N. N., Gilmore, A., Gillespie, R. J., Salata, M. J., Liu, R. W., & Voos, J. E. (2019). A 46-year Analysis of Gender Trends in Academic Authorship in Orthopaedic Sports Medicine. *J Am Acad Orthop Surg*, *27*(13), 493-501. <https://doi.org/10.5435/JAAOS-D-18-00669>
55. Kongkiatkamon, S., Yuan, J. C., Lee, D. J., Knoernschild, K. L., Campbell, S. D., & Sukotjo, C. (2010). Gender disparities in prosthodontics: authorship and leadership, 13 years of observation. *J Prosthodont*, *19*(7), 565-570. <https://doi.org/10.1111/j.1532-849X.2010.00620.x>
56. Labinaz, A., Marbach, J. A., Jung, R. G., Moreland, R., Motazedian, P., Di Santo, P., Clancy, A. A., MacDonald, Z., Simard, T., Hibbert, B., & Ramirez, F. D. (2019). Female Authorship in Preclinical Cardiovascular Research: Temporal Trends and Influence on Experimental Design. *JACC Basic Transl Sci*, *4*(4), 471-477. <https://doi.org/10.1016/j.jacbts.2019.04.004>
57. Lasnon, C., Girault, G., Lebtahi, R., Ansquer, C., Lequesne, J., & Quak, E. (2021). Women Authors in Nuclear Medicine Journals: a Survey from 2014 to 2020. *Journal of nuclear medicine : official publication, Society of Nuclear Medicine*. <https://doi.org/10.2967/jnumed.121.262773>
58. Lennon, M. J., Kennedy, R., Ryan, H., Neuen, D. R., & Godwin, M. (2021). Changes in the proportions of authors in Australian medical journals who were women, 2005-2018. *Medical Journal of Australia*, *214*, 376-377. <https://doi.org/10.5694/mja2.50998>
59. Lennox, M. G., Li, M., Wang, X., Pien, L. C., & Lang, D. M. (2019). Patterns of North American Women Authorship in 2 Allergy/Immunology Journals: 1997-2017. *J Allergy Clin Immunol Pract*, *7*(7), 2156-2160. <https://doi.org/10.1016/j.jaip.2019.03.040>
60. Lerchenmuller, C., Schmallenbach, L., Jena, A. B., & Lerchenmueller, M. J. (2021). Longitudinal analyses of gender differences in first authorship publications related to COVID-19. *BMJ OPEN*, *11*(4). <https://doi.org/10.1136/bmjopen-2020-045176>
61. Leung, K. K., Jawaid, N., & Bollegala, N. (2021). Gender differences in gastroenterology and hepatology authorship and editorial boards. *Gastrointestinal Endoscopy*, *94*(4), 713-723. <https://doi.org/10.1016/j.gie.2021.05.019>
62. Liang, T., Zhang, C., Khara, R. M., & Harris, A. C. (2015). Assessing the Gap in Female Authorship in Radiology: Trends Over the Past Two Decades. *J Am Coll Radiol*, *12*(7), 735-741. <https://doi.org/10.1016/j.jacr.2015.03.008>
63. Long, M. T., Leszczynski, A., Thompson, K. D., Wasan, S. K., & Calderwood, A. H. (2015). Female authorship in major academic gastroenterology journals: a look over 20 years [Article]. *Gastrointestinal Endoscopy*, *81*(6), 1440-+. <https://doi.org/10.1016/j.gie.2015.01.032>
64. Luc, J. G. Y., Vervoort, D., Percy, E., Hirji, S., Mann, G. K., Phan, K., Dibas, M., Vaduganathan, M., Preventza, O., & Antonoff, M. B. (2021). Trends in Female Authorship: A Bibliometric Analysis of The Annals of Thoracic Surgery [Research Support, N.I.H., Extramural]. *Annals of Thoracic Surgery*, *111*(4), 1387-1393.
65. Lydon, S., Madden, C., De Bhulbh, A., Maher, S., Byrne, D., & O'Connor, P. (2021). Do Gender-Based Disparities in Authorship // Editorship Exist in Healthcare Simulation Journals? A Bibliometric Review of the Research. *SIMULATION IN HEALTHCARE-JOURNAL OF THE SOCIETY FOR SIMULATION IN HEALTHCARE*, *16*(2). <https://doi.org/10.1097/SIH.0000000000000453>
66. Madden, C., O'Malley, R., O'Connor, P., O'Dowd, E., Byrne, D., & Lydon, S. (2021). Gender in authorship and editorship in medical education journals: A bibliometric review. *Medical Education*, *55*(6), 678-688. <https://doi.org/10.1111/medu.14427>
67. Mahajan, U. V., Wadhwa, H., Fatemi, P., Xu, S., Shan, J., Benzil, D. L., & Zygourakis, C. C. (2020). Does double-blind peer review impact gender authorship trends? An evaluation of two leading neurosurgical journals from 2010 to 2019. *Journal of neurosurgery*. <https://doi.org/10.3171/2020.6.JNS20902>
68. Malchuk, A. M., Coffman, M., Wilkinson, E., & Jabbarpour, Y. (2021). Gender Concordance of First and Senior Authors in Family Medicine Journals. *Family Medicine*, *53*(2), 92-97.
69. Mamtani, M., Shofer, F., Mudan, A., Khatri, U., Walker, R., Perrone, J., & Aysola, J. (2020). Quantifying gender disparity in physician authorship among commentary articles in three high-impact medical journals: an observational study [Observational Study]. *BMJ Open*, *10*(2), e034056.
70. Manlove, K. R., & Belou, R. M. (2018). Authors and editors assort on gender and geography in high-rank ecological publications. *PLoS One*, *13*(2), e0192481. <https://doi.org/10.1371/journal.pone.0192481>
71. Mansour, A. M., Shields, C. L., Maalouf, F. C., Massoud, V. A., Jurdy, L., Mathysen, D. G., Jaafar, D., & Aclimandos, W. (2012). Five-decade profile of women in leadership positions at ophthalmic publications. *Arch Ophthalmol*, *130*(11), 1441-1446. <https://doi.org/10.1001/archophthalmol.2012.2300>
72. Marrone, A. F., Berman, L., Brandt, M. L., & Rothstein, D. H. (2020). Does academic authorship reflect gender bias in pediatric surgery? An analysis of the Journal of Pediatric Surgery, 2007-2017. *Journal of Pediatric Surgery*, *55*(10), 2071-2074.
73. Matchanova, A., Avci, G., Babicz, M. A., Thompson, J. L., Johnson, B., Ke, I. J., Rahman, S., Sullivan, K. L., Sheppard, D. P., Morales, Y., Tierney, S. M., Kordovski, V. M., Beltran-Najera, I., Ulrich, N., Pilloff, S., Yeates, K. O., & Woods, S. P. (2020). Gender disparities in the author bylines of articles published in clinical neuropsychology journals from 1985 to 2019. *CLINICAL NEUROPSYCHOLOGIST*. <https://doi.org/10.1080/13854046.2020.1843713>
74. Mehta, A. K., Patel, R., Patel, D., & Davis, M. P. (2021). Trends in Published Palliative Care Research: A 15-Year Review. *American Journal of Hospice & Palliative Medicine*, *38*(5), 489-493. <https://doi.org/10.1177/1049909120944863>
75. Mendlowicz, M. V., Coutinho, E. S. F., Laks, J., Fontenelle, L. F., Valença, A. M., Berger, W., Figueira, I., & de Aguiar, G. A. (2011). Is there a ‘gender gap’ in authorship of the main Brazilian psychiatric journals at the beginning of the 21st century? *Scientometrics*, *86*(1), 27-37. <https://doi.org/10.1007/s11192-010-0296-3>
76. Merriman, R., Galizia, I., Tanaka, S., Sheffel, A., Buse, K., & Hawkes, S. (2021). The gender and geography of publishing: a review of sex/gender reporting and author representation in leading general medical and global health journals. *BMJ global health*, *6*(5). <https://doi.org/10.1136/bmjgh-2021-005672>
77. Miller, A. L., Rathi, V. K., Gray, S. T., & Bergmark, R. W. (2020). Female Authorship of Opinion Pieces in Leading Otolaryngology Journals between 2013 and 2018. *Otolaryngology - Head & Neck Surgery*, *162*(1), 35-37.
78. Miller, J., Chuba, E., Deiner, S., DeMaria, S., Jr., & Katz, D. (2019). Trends in Authorship in Anesthesiology Journals. *Anesth Analg*, *129*(1), 306-310. <https://doi.org/10.1213/ANE.0000000000003949>
79. Molwitz, I., Yamamura, J., Ozga, A.-K., Wedekind, I., Nguyen, T.-A., Wolf, L., Kamo, M., Zhao, J., Can, E., & Keller, S. (2021). Gender trends in authorships and publication impact in Academic Radiology-a 10-year perspective. *EUROPEAN RADIOLOGY*. <https://doi.org/10.1007/s00330-021-07928-4>
80. Muric, G., Lerman, K., & Ferrara, E. (2021). Gender Disparity in the Authorship of Biomedical Research Publications During the COVID-19 Pandemic: Retrospective Observational Study. *JMIR* <https://doi.org/10.2196/25379>
81. Nguyen, A. X., Trinh, X.-V., Kurian, J., & Wu, A. Y. (2021). Impact of COVID-19 on longitudinal ophthalmology authorship gender trends. *Graefe's Archive for Clinical and Experimental Ophthalmology*. <http://dx.doi.org/10.1007/s00417-021-05085-4>
82. Okafor, S., Tibbetts, K., Shah, G., Tillman, B., Agan, A., & Halderman, A. A. (2020). Is the gender gap closing in otolaryngology subspecialties? An analysis of research productivity. *Laryngoscope*, *130*(5), 1144-1150. <https://doi.org/10.1002/lary.28189>
83. Ouyang, D., Sing, D., Shah, S., Hu, J., Duvernoy, C., Harrington, R. A., & Rodriguez, F. (2018). Sex Disparities in Authorship Order of Cardiology Scientific Publications. *Circ Cardiovasc Qual Outcomes*, *11*(12), e005040. <https://doi.org/10.1161/CIRCOUTCOMES.118.005040>
84. Pagel, P. S., Freed, J. K., & Lien, C. A. (2019). Gender Differences in Authorship in the Journal of Cardiothoracic and Vascular Anesthesia: A 28-Year Analysis of Publications Originating From the United States, 1990-2017. *J Cardiothorac Vasc Anesth*, *33*(3), 593-599. <https://doi.org/10.1053/j.jvca.2018.11.017>
85. Pasick, L. J., Yeakel, H., & Sataloff, R. T. (2020). Sex Bias in Laryngology Research and Publishing. *Journal of voice : official journal of the Voice Foundation*. <https://doi.org/10.1016/j.jvoice.2020.06.021>
86. Pastor-Cabeza, M., Torne, R., Garcia-Armengol, R., Menendez-Osorio, B., Mosteiro-Cadaval, A., Bollar, A., Rimbau, J. M., Sarabia, R., & Rodriguez-Hernandez, A. (2021). Women's role in neurosurgical research: is the gender gap improving? *Neurosurgical Focus*, *50*(3), E6.
87. <https://thejns.org/downloadpdf/journals/neurosurg-focus/50/3/article-pE6.pdf>
88. Pico T, Bierman T, Doyle K, & Richardson, S. (2020). First Authorship Gender Gap in the Geosciences. *Earth and Space Science*. <https://doi.org/10.1029/2020EA001203>
89. Piper, C. L., Scheel, J. R., Lee, C. I., & Forman, H. P. (2016). Gender Trends in Radiology Authorship: A 35-Year Analysis. *AJR Am J Roentgenol*, *206*(1), 3-7. <https://doi.org/10.2214/AJR.15.15116>
90. Polanco, N. A. P., McNally, B. B., Levy, C., Carey, E. J., Palomique, J., & Tran, T. T. (2020). Gender Differences in Hepatology Medical Literature. *Dig Dis Sci*, *65*(10), 3014-3022. <https://doi.org/10.1007/s10620-019-06025-3>
91. Prior, A., Ogburu-Ogbonnaya, N., Barfield, W. R., Mooney Iii, J. F., Van Nortwick, S., Murphy, R. F., & Mooney, J. F., 3rd. (2021). Analysis of Author Gender in the Pediatric Orthopaedic Literature from 2011 to 2020. *Journal of Pediatric Orthopaedics*, *41*(7), e481-e483. <https://doi.org/10.1097/BPO.0000000000001844>
92. Purdy, M. E., Zmuda, B. N., Owens, A. M., Choudhary, V., Olsen, R. C., Bader, J. O., & Donaldson, C. M. (2021). Gender differences in publication in emergency medicine journals. *The American journal of emergency medicine*, *49*. <https://doi.org/10.1016/j.ajem.2021.06.039>
93. Pyatigorskaya, N., & Di Marco, L. (2017). Women authorship in radiology research in France: An analysis of the last three decades. *Diagn Interv Imaging*, *98*(11), 769-773. <https://doi.org/10.1016/j.diii.2017.07.001>
94. Qureshi, R., Han, G., Fapohunda, K., Abariga, S., Wilson, R., & Li, T. (2020). Authorship diversity among systematic reviews in eyes and vision [Research Support, N.I.H., Extramural]. *Systematic Reviews*, *9*(1), 192.
95. Ravi, K., Bentounsi, Z., Tariq, A., Brazeal, A., Daudu, D., Back, F., Elhadi, M., Badwi, N., Shah, S. S. N. H., Bandyopadhyay, S., Khalil, H., Kimura, H., Sekyi-Djan, M. N., Abdelrahman, A., Shaheen, A., Mbonda Noula, A. G., Wong, A.-T., Ndajiwo, A., Souadka, A., . . . Sharma, D. (2021). Systematic analysis of authorship demographics in global surgery. *BMJ global health*, *6*(10). <https://doi.org/10.1136/bmjgh-2021-006672>
96. Rickard, M., Hannick, J. H., Blais, A.-S., Wang, J., Santos, J. D., & Lorenzo, A. J. (2020). Female Authorship Publishing Trends and Forecasting in Pediatric Urology: Are We Closer to Gender Equality? *Urology*, *139*, 141-150. <https://doi.org/10.1016/j.urology.2020.01.037>
97. Rock, K. N., Barnes, I. N., Deyski, M. S., Glynn, K. A., Milstead, B. N., Rottenborn, M. E., Andre, N. S., Dekhtyar, A., Dekhtyar, O., & Taylor, E. N. (2021). Quantifying the Gender Gap in Authorship in Herpetology. *HERPETOLOGICA*, *77*(1). <https://doi.org/10.1655/0018-0831-77.1.1>
98. Russell, A. F., Loder, R. T., Gudeman, A. S., Bolaji, P., Virtanen, P., Whipple, E. C., & Kacena, M. A. (2019). A Bibliometric Study of Authorship and Collaboration Trends Over the Past 30 Years in Four Major Musculoskeletal Science Journals. *Calcif Tissue Int*, *104*(3), 239-250. <https://doi.org/10.1007/s00223-018-0492-3>
99. Rynecki, N. D., Krell, E. S., Potter, J. S., Ranpura, A., & Beebe, K. S. (2020). How Well Represented Are Women Orthopaedic Surgeons and Residents on Major Orthopaedic Editorial Boards and Publications? *Clinical Orthopaedics & Related Research*, *478*(7), 1563-1568.
100. Salinaro, J. R., Puechl, A. M., Havrilesky, L. J., & Davidson, B. A. (2018). Gender trends in gynecologic oncology authorship: Implications for the critical evaluation of gender distribution in academic rank and leadership positions. *Gynecol Oncol*, *151*(3), 542-546. <https://doi.org/10.1016/j.ygyno.2018.10.009>
101. Schauwecker, N., Kaplan, A., & Hunter, J. B. (2021). Gender Prevalence and Trends in Otology and Neurotology Publications. *Otology & Neurotology*, *42*(5), 659-665. <https://doi.org/10.1097/MAO.0000000000003067>
102. Schrager, S., Bouwkamp, C., & Mundt, M. (2011). Gender and first authorship of papers in family medicine journals 2006--2008. *Fam Med*, *43*(3), 155-159. <https://www.ncbi.nlm.nih.gov/pubmed/21380946>
103. Sela, N., Anderson, B. L., Granatowicz, A. T., Jezewski, E., & Hoffman, A. L. (2021). Gender differences in authorship among Hepato- pancreatico-biliary surgeons. *HPB*, *23*(6). <https://doi.org/10.1016/j.hpb.2020.11.1151>
104. Shah, D. N., Huang, J., Ying, G. S., Pietrobon, R., & O'Brien, J. M. (2013). Trends in female representation in published ophthalmology literature, 2000-2009. *Digit J Ophthalmol*, *19*(4), 50-55. <https://doi.org/10.5693/djo.01.2013.07.002>
105. Shah, S. G. S., Dam, R., Milano, M. J., Edmunds, L. D., Henderson, L. R., Hartley, C. R., Coxall, O., Ovseiko, P. V., Buchan, A. M., & Kiparoglou, V. (2021). Gender parity in scientific authorship in a National Institute for Health Research Biomedical Research Centre: a bibliometric analysis [Research Support, Non-U.S. Gov't]. *BMJ Open*, *11*(3), e037935.
106. Sheridan, G., Wisken, E., Hing, C. B., & Smith, T. O. (2018). A bibliometric analysis assessing temporal changes in publication and authorship characteristics in The Knee from 1996 to 2016. *Knee*, *25*(2), 213-218. <https://doi.org/10.1016/j.knee.2018.01.014>
107. Shukla, D. C., Simma-Chiang, V., Kyprianou, N., Tewari, A. K., & Lundon, D. J. (2021). Does Gender Matter in Academic Surgery? Author and Mentor Gender Impact Publication Citations in Surgical Research. *Urology*. <https://doi.org/10.1016/j.urology.2021.04.049>
108. Sidhu, R., Rajashekhar, P., Lavin, V. L., Parry, J., Attwood, J., Holdcroft, A., & Sanders, D. S. (2009). The gender imbalance in academic medicine: a study of female authorship in the United Kingdom. *J R Soc Med*, *102*(8), 337-342. <https://doi.org/10.1258/jrsm.2009.080378>
109. Sing, D. C., Jain, D., & Ouyang, D. (2017). Gender trends in authorship of spine-related academic literature-a 39-year perspective. *Spine J*, *17*(11), 1749-1754. <https://doi.org/10.1016/j.spinee.2017.06.041>
110. Skinner, C. H., Robinson, S. L., Brown, C. S., & Cates, G. L. (1999). Female publication patterns in School Psychology Review, Journal of School Psychology, and School Psychology Quarterly from 1985-1994 [Article]. *School Psychology Review*, *28*(1), 76-83. <Go to ISI>://WOS:000079675200007
111. Sotudeh, H., Dehdarirad, T., & Freer, J. (2018). Gender differences in scientific productivity and visibility in core neurosurgery journals: Citations and social media metrics [Article]. *Research Evaluation*, *27*(3), 262-269. <https://doi.org/10.1093/reseval/rvy003>
112. Stinson, S. (2003). Participation of women in human biology, 1975-2001. *Am J Hum Biol*, *15*(3), 440-445. <https://doi.org/10.1002/ajhb.10160>
113. Strand, M., & Bulik, C. M. (2018). Trends in female authorship in research papers on eating disorders: 20-year bibliometric study. *BJPsych Open*, *4*(2), 39-46. <https://doi.org/10.1192/bjo.2017.8>
114. Subramaniam, M., Azad, N., Wasan, S. K., & Long, M. T. (2021). Equal Opportunity: Women Representation on Editorial Boards and Authorship of Editorials in Gastroenterology and Hepatology Journals. *The American journal of gastroenterology*, *116(3)*, 613-616.
115. Sussenbacher, S., Amering, M., Gmeiner, A., & Schrank, B. (2017). Gender-gaps and glass ceilings: A survey of gender-specific publication trends in Psychiatry between 1994 and 2014 [Article]. *European Psychiatry*, *44*, 90-95. <https://doi.org/10.1016/j.eurpsy.2017.03.008>
116. Swarnkar, P., Sinha, V., Spake, C., Crozier, J., Ngaage, L. M., Roussel, L. O., & Borrelli, M. R. (2021). Women in Cosmetic Plastic Surgery: An Analysis of Female Authorship in Cosmetic Plastic Surgery Over the Last 10 Years. *The American journal of Cosmetic Surgery*. <http://dx.doi.org/10.1177/0748806821991416>
117. Taha, B., Sadda, P., Winston, G., Odigie, E., Londono, C., Greenfield, J. P., Pannullo, S. C., & Hoffman, C. (2021). Increases in female academic productivity and female mentorship highlight sustained progress in previously identified neurosurgical gender disparities [Meta-Analysis]. *Neurosurgical Focus*, *50*(3), E3.
118. Takayanagui, O. M., & Livramento, J. A. (2009). The increasing female participation in authorship of articles published in neurology in Brazil. *Arq Neuropsiquiatr*, *67*(3B), 914-916. <https://doi.org/10.1590/s0004-282x2009000500029>
119. Terry, J. L. (1996). Authorship in College & Research Libraries revisited: Gender, institutional affiliation, collaboration [Article]. *College & Research Libraries*, *57*(4), 377-383. <https://doi.org/10.5860/crl_57_04_377>
120. Thomson, A., Horne, R., Chung, C., Marta, M., Giovannoni, G., Palace, J., & Dobson, R. (2019). Visibility and representation of women in multiple sclerosis research. *Neurology*, *92*(15), 713-719. <https://doi.org/10.1212/WNL.0000000000007276>
121. van Doren, S., Brida, M., Gatzoulis, M. A., Kempny, A., Babu-Narayan, S. V., Bauer, U. M. M., Baumgartner, H., & Diller, G. P. (2019). Sex differences in publication volume and quality in congenital heart disease: are women disadvantaged? *Open Heart*, *6*(1), e000882. <https://doi.org/10.1136/openhrt-2018-000882>
122. Vasti, E. C., Ouyang, D., Ngo, S., Sarraju, A., Harrington, R. A., & Rodriguez, F. (2021). Gender Disparities in Cardiology-Related COVID-19 Publications. *CARDIOLOGY // THERAPY*. <https://doi.org/10.1007/s40119-021-00234-6>
123. Vela, B., Caceres, P., & Cavero, J. M. (2012). Participation of women in software engineering publications [Article]. *Scientometrics*, *93*(3), 661-679. <https://doi.org/10.1007/s11192-012-0774-x>
124. Vranas, K. C., Ouyang, D., Lin, A. L., Slatore, C. G., Sullivan, D. R., Kerlin, M. P., Liu, K. D., Baron, R. M., Calfee, C. S., Ware, L. B., Halpern, S. D., Matthay, M. A., Herridge, M. S., Mehta, S., & Rogers, A. J. (2020). Gender Differences in Authorship of Critical Care Literature. *AMERICAN JOURNAL OF RESPIRATORY // CRITICAL CARE MEDICINE*, *201*(7). <https://doi.org/10.1164/rccm.201910-1957OC>
125. Wang, S. C., Koutroumpakis, E., Schulman-Marcus, J., Tosh, T., Volgman, A. S., & Lyubarova, R. (2021). Sex Differences Remain Under-Reported in Cardiovascular Publications. *Journal of Women's Health (15409996)*, *30*(9), 1253-1258. <https://doi.org/10.1089/jwh.2020.8561>
126. Webb, J., Cambron, J., Xu, K. T., Simmons, M., & Richman, P. (2021). First and last authorship by gender in emergency medicine publications- a comparison of 2008 vs. 2018. *American Journal of Emergency Medicine*, *46*, 445-448. <https://doi.org/10.1016/j.ajem.2020.10.045>
127. West, J. D., Jacquet, J., King, M. M., Correll, S. J., & Bergstrom, C. T. (2013). The role of gender in scholarly authorship. *PLoS One*, *8*(7), e66212. <https://doi.org/10.1371/journal.pone.0066212>
128. Whitley, J. A., Holt, S. K., Nelson, D., & Kieran, K. (2021). Gender Differences in Authorship in Urology: a Five-year Review of Publications in Five High-impact Journals. *Urology*, *150*, 9-15. <https://doi.org/10.1016/j.urology.2020.07.080>
129. Williams, E. A., Kolek, E. A., Saunders, D. B., Remaly, A., & Wells, R. S. (2018). Mirror on the Field: Gender, Authorship, and Research Methods in Higher Education's Leading Journals [Article]. *Journal of Higher Education*, *89*(1), 28-53. <https://doi.org/10.1080/00221546.2017.1330599>
130. Williams, W. A., 2nd, Li, A., Goodman, D. M., & Ross, L. F. (2021). Impact of the Coronavirus Disease 2019 Pandemic on Authorship Gender in The Journal of Pediatrics: Disproportionate Productivity by International Male Researchers. *Journal of Pediatrics*, *231*, 50-54.
131. Wilson, D. S. (1998). Patterns in publishing in three North American herpetological journals: Gender biases [Article]. *Herpetologica*, *54*, S35-S42. <Go to ISI>://WOS:000074219600008
132. Xiao, N., Oliveira, D. F. M., & Gupta, R. (2018). Characterizing the Impact of Women in Academic IR: A 12-Year Analysis. *J Vasc Interv Radiol*, *29*(11), 1553-1557. <https://doi.org/10.1016/j.jvir.2018.06.010>
133. Xu, R. F., Varady, N. H., & Chen, A. F. (2020). Trends in Gender Disparities in Authorship of Arthroplasty Research. *Journal of Bone & Joint Surgery, American Volume*, *102*(23), 1-8. <https://doi.org/10.2106/JBJS.20.00258>
134. Yalamanchali, A., Zhang, E. S., & Jagsi, R. (2021). Trends in Female Authorship in Major Journals of 3 Oncology Disciplines, 2002-2018. *JAMA Network Open*, *4*(4), e212252-e212252. <https://doi.org/10.1001/jamanetworkopen.2021.2252>
135. Yuan, J. C., Lee, D. J., Kongkiatkamon, S., Ross, S., Prasad, S., Koerber, A., & Sukotjo, C. (2010). Gender trends in dental leadership and academics: a twenty-two-year observation. *J Dent Educ*, *74*(4), 372-380. <https://www.ncbi.nlm.nih.gov/pubmed/20388809>
136. Yun, E. J., Yoon, D. Y., Kim, B., Moon, J. Y., Yoon, S. J., Hong, S. J., & Baek, S. (2015). Closing the Gender Gap: Increased Female Authorship in AJR and Radiology. *AJR Am J Roentgenol*, *205*(2), 237-241. <https://doi.org/10.2214/AJR.14.14225>
137. Zehetbauer, R., von Haugwitz, F., & Seifert, R. (2021). Gender-specific analysis of the authors and the editorial board of Naunyn-Schmiedeberg's Archives of Pharmacology from 2000 to 2020. Naunyn-Schmiedeberg's Archives of Pharmacology. <https://doi.org/10.1007/s00210-021-02166-3>
